# Blood transcriptome responses in patients correlate with severity of COVID-19 disease

**DOI:** 10.3389/fimmu.2022.1043219

**Published:** 2023-01-20

**Authors:** Ya Wang, Klaus Schughart, Tiana Maria Pelaia, Tracy Chew, Karan Kim, Thomas Karvunidis, Ben Knippenberg, Sally Teoh, Amy L. Phu, Kirsty R. Short, Jonathan Iredell, Irani Thevarajan, Jennifer Audsley, Stephen Macdonald, Jonathon Burcham, Anthony McLean, PREDICT-19 consortium, Alberto Ballestrero, Benjamin Tang, Maryam Shojaei

**Affiliations:** ^1^ Department of Intensive Care Medicine, Nepean Hospital, Penrith, NSW, Australia; ^2^ Centre for Immunology and Allergy Research, The Westmead Institute for Medical Research, Westmead, NSW, Australia; ^3^ Faculty of Medicine and Health, Sydney Medical School Nepean, Nepean Hospital, University of Sydney, Penrith, NSW, Australia; ^4^ Department of Microbiology, Immunology and Biochemistry, University of Tennessee Health Science Center, Memphis, TN, United States; ^5^ Institute of Molecular Virology, University of Münster, Münster, Germany; ^6^ Sydney Informatics Hub, Core Research Facilities, The University of Sydney, Sydney, NSW, Australia; ^7^ Medical ICU, 1^st^ Department of Internal Medicine, Charles University and Teaching Hospital, Pilsen, Czechia; ^8^ Department of Microbiology. St George Hospital, Kogarah, NSW, Australia; ^9^ Research and Education Network, Western Sydney Local Health District, Westmead Hospital, NSW, Westmead, Australia; ^10^ Faculty of Medicine and Health, Sydney Medical School Westmead, Westmead Hospital, University of Sydney, NSW, Westmead, Australia; ^11^ School of Chemistry and Molecular Biosciences, The University of Queensland, Brisbane, QLD, Australia; ^12^ Centre for Infectious Diseases and Microbiology, The Westmead Institute for Medical Research, Westmead, NSW, Australia; ^13^ Faculty of Medicine and Health, School of Medical Sciences, The University of Sydney, Sydney, NSW, Australia; ^14^ Westmead Hospital, Western Sydney Local Health District, Westmead, NSW, Australia; ^15^ Sydney Institute for Infectious Disease, The University of Sydney, Sydney, NSW, Australia; ^16^ Victorian Infectious Disease Service, The Royal Melbourne Hospital at the Peter Doherty Institute for Infection and Immunity, Melbourne, VIC, Australia; ^17^ Department of Infectious Diseases, The University of Melbourne at the Peter Doherty Institute for Infection and Immunity, Melbourne, VIC, Australia; ^18^ Centre for Clinical Research in Emergency Medicine, Harry Perkins Institute of Medical Research, Royal Perth Hospital, Perth, WA, Australia; ^19^ Medical School, University of Western Australia, Perth, WA, Australia; ^20^ Emergency Department, Royal Perth Hospital, Perth, WA, Australia; ^21^ Centre for Clinical Research in Emergency Medicine, Royal Perth Bentley Group, Perth, WA, Australia

**Keywords:** SARS-CoV-2, RNA sequencing, host immune response, deconvolution, WGCNA

## Abstract

**Background:**

Coronavirus disease 2019 (COVID-19) is an infectious disease caused by severe acute respiratory syndrome coronavirus 2 (SARS-CoV-2). Infected individuals display a wide spectrum of disease severity, as defined by the World Health Organization (WHO). One of the main factors underlying this heterogeneity is the host immune response, with severe COVID-19 often associated with a hyperinflammatory state.

**Aim:**

Our current study aimed to pinpoint the specific genes and pathways underlying differences in the disease spectrum and outcomes observed, through in-depth analyses of whole blood transcriptomics in a large cohort of COVID-19 participants.

**Results:**

All WHO severity levels were well represented and mild and severe disease displaying distinct gene expression profiles. WHO severity levels 1-4 were grouped as mild disease, and signatures from these participants were different from those with WHO severity levels 6-9 classified as severe disease. Severity level 5 (moderate cases) presented a unique transitional gene signature between severity levels 2-4 (mild/moderate) and 6-9 (severe) and hence might represent the turning point for better or worse disease outcome. Gene expression changes are very distinct when comparing mild/moderate or severe cases to healthy controls. In particular, we demonstrated the hallmark down-regulation of adaptive immune response pathways and activation of neutrophil pathways in severe compared to mild/moderate cases, as well as activation of blood coagulation pathways.

**Conclusions:**

Our data revealed discrete gene signatures associated with mild, moderate, and severe COVID-19 identifying valuable candidates for future biomarker discovery.

## Introduction

Since December 2019, COVID-19 has become a major global health concern with 6.3 million deaths recorded globally as of 11 July 2022 (https://covid19.who.int). Infected patients have various disease manifestations and trajectories, which poses a challenge to patient management and resource planning. Initial findings from 44,672 confirmed COVID-19 cases published by the Chinese Center for Disease Control and Prevention in early 2020, identified 3 groups of patients: 1) 81% patients had only mild symptoms (e.g., fever, cough, fatigue, muscle pain, etc.) with no or mild pneumonia; 2) 14% patients had severe symptoms with dyspnea and hypoxia (blood oxygen saturation ≤ 93%); 3) 5% patients had critical symptoms with respiratory failure, septic shock, and/or multiple organ dysfunction (1). Later in June 2020, WHO published a more refined ordinal scale for grading patient severity, clinical progression and recovery based on the level of care required and the need for supportive measures (2). The scale consists of 11 levels: 0 (uninfected) to 10 (death), with levels 1-3 classified as mild disease without the need of hospitalization, levels 4-5 as hospitalized moderate disease with or without non-invasive supplemental oxygen administration, and levels 6-9 as hospitalized severe disease requiring mechanical ventilation and/or intubation. Mild disease tends to be self-limiting. For patients with moderate or severe disease requiring medical care, accurate diagnostic and prognostic tools are crucial for resource planning and management.

Numerous studies have uncovered the underlying immunological characteristics associated with the mild, moderate, and severe diseases at both transcriptomic and proteomic levels, which provide basis for better patient triage, monitoring patients’ response to treatment and designing new treatments. Compared to the mild or moderate category, immune dysregulation is evident in the severe category with elevated serum levels of proinflammatory cytokines (in particularly, interleukin-6 (IL6) and tumor necrosis factor alpha (TNF)), C-reactive protein (CRP), and higher neutrophil/lymphocyte ratio (3–5). Further evidence reveals that immune dysregulation in severe COVID-19 is characterized by: 1) impaired or delayed type I interferon (IFN-I) response (6–10); 2) aberrant activation and enrichment of neutrophils (11–13) and; 3) lymphopenia (14–16). As a result, severe COVID-19 is associated with a hyperinflammatory state with diminished anti-viral response. As compared to the patients with mild to moderate diseases, main effectors of the IFN-I mediated anti-viral response, interferon-stimulated genes (ISGs) (e.g., *IFI44L, IFI27, RSAD2, SIGLEC1, IFIT1, ISG15*) are downregulated in the critical patients (6). IFN-I, on the other hand, triggers production of proinflammatory cytokines through nuclear factor-κB (NF- κB) signaling, contributing to the hyperinflammatory state in severe COVID-19 (9). Neutrophil activation cytokines such as IL-8 and granulocyte colony-stimulating factor (G-CSF), together with neutrophil-derived effectors such as resistin (RETN), lipocalin-2 (LCN2), and hepatocyte growth factor (HGF), are elevated in the plasma from the severe COVID-19 patients compared to the mild/moderate patients (11, 12). Transcriptomic analyses also reveal significant upregulation of genes involved in neutrophil activation in the severe patients compared to the mild ones, including *CD177*, matrix metalloproteinases 8 and 9 (*MMP8* and *MMP9*), neutrophil elastase (*ELANE*), olfactomedin 4 (*OLFM4*), myeloperoxidase (*MPO*), and alarmins (*S100A8, 9, and 12*) (13). Lymphopenia with blood lymphocyte count < 1 x 10^9^/L, is observed in most patients with severe COVID-19. Decreased blood lymphocyte percentage over time is associated with poor prognosis and recovery of patients from COVID-19 has been associated with restored blood lymphocyte percentage (14, 16, 17). Lymphopenia in COVID-19 is particularly related to the loss of CD4^+^ and CD8^+^ T cells (18), which can be caused by 1) overproduction of proinflammatory cytokines IL-6 (3); 2) overexpression of T cell exhaustion markers programmed cell death protein 1 (PD-1) and T cell immunoglobulin and mucin domain 3 (TIM-3) (19); 3) presence of a suppressive neutrophil subset known as granulocytic myeloid-derived suppressor cells (G-MDSC) and its production of alarmin S100A8 and A9 (20).

These studies provide important evidence about the association of host immune responses with disease severity and outcome. However, how each severity level within the category differs from the others at transcriptomic level is not well studied. Here, we analyzed the whole blood transcriptomics by RNA sequencing (RNASeq), from COVID-19 patients with disease severity levels ranging from 0-9. In-depth DEG, deconvolution, correlation and WGCNA analyses of the RNASeq data were performed to compare between individual severity level and between severity categories. Our results corroborate findings from previous studies as above. Most importantly, we discovered a transitional level between severity levels 2-4 (mild/moderate) and 6-9 (severe). Further dissection of this transitional group might unravel the mechanism underlying disease progression of COVID-19 from moderate to severe.

## Materials and methods

### Study design and participants of human cohorts

In this study, participants were recruited from multiple centers from Sydney, Melbourne, and Perth in Australia and a single center in Czech Republic between February 2020 and February 2021. Eligibility criteria included (1) age equal or greater than 18 years, (2) World Health Organization definition of influenza-like illness (fever of 38 C° or higher, cough, sore throat, nasal congestion, and illness onset within the last ten days), and (3) Participants with SARS-CoV-2 infection confirmed by virological testing- respiratory samples (nasal/throat swab/sputum/bronchoalveolar lavage) collected from participants and tested for SARS-CoV-2 virus. All eligible participants were assessed by an admitting physician for likelihood of infection. Participants with a high likelihood of infection, based on history and clinical features, were also enrolled into the study. Seventy-one healthy volunteer’s samples included in this study were all collected prior 2018. Study data were collected and managed using REDCap electronic data capture tools (21, 22) hosted at the University of Sydney.

### Blood sample collection and RNA isolation

Two and half millilitres of blood was collected into PAXgene Blood RNA tubes (Qiagen) from participants according to the manufacturer’s supplied protocol, resulting in a total of 203 samples (multiple samples were taken from some participants). Collected samples were invert 8–10 times gently, immediately after blood collection, kept for ~2h at room temperature, followed by incubation at -20°C for 24h. Thereafter tubes were transferred to -80°C prior to processing. Total RNA was isolated from whole blood samples stored and stabilized in PAXgene RNA tubes according to the manufacturer’s guidelines (PreAnalytiX). The quality and quantity of extracted RNA was evaluated by visualization of 28S and 18S band integrity on a Tapestation 4200 system (Agilent) and stored at -80°C.

### Library preparation and RNASeq

Libraries were prepared with 300ng of total RNA per sample using the Illumina Stranded Total RNA Prep with Ribo-Zero Plus (RZP) as per manufacturer instructions (Illumina, CA, USA). Final libraries were cleaned using beads (Beckman Coulter, IN, USA), quantified, and normalised with qPCR using NEBNext Library Quant Kit for Illumina. All libraries were pooled with 32 samples per lane and sequenced with 150 bp paired-end (PE) reads using an Illumina NovaSeq 6000 with v1.5 chemistry and S4-300 flow cell. A minimum sequencing depth of 48.3 million (M) read pairs were generated from each library. Base calling and FASTQ conversion were complete with NovaSeq Control Software (NCS) v1.7.5, Real Time Analysis (RTA) v3.4.4 and Illumina DRAGEN BCL Convert 07.021.624.3.10.8. FASTQ files were uploaded into Partek Flow software (Partek Inc., MO, USA), and primary QC was performed.

### Bioinformatic analysis of RNASeq data

FASTQ files containing raw sequencing data were quality controlled and pre-processed into analysis ready count data using the highly scalable RNASeq-DE workflow, available online at https://github.com/Sydney-Informatics-Hub/RNASeq-DE (v1.0.0) (23). Default settings were applied unless otherwise described here. Briefly, 3’ adapter and polyA tails were trimmed from raw sequence reads with BBDuk (v37.89) (24). An average of 89.2 million trimmed reads per sample were remaining. FastQC (v0.11.7) (25) was used to confirm that median sequence and base qualities scored Phred > 20. Quality checked, trimmed reads were aligned as pairs to the human reference genome, GRCh38 primary assembly and gene set release 106 (obtained from Ensembl) with STAR, setting –sjdbOverhand to 149. Sequencing batch level binary alignment (BAM) files were merged and indexed with SAMtools (v1.10) (26) to obtain sample level BAMs. HTSeq-count (v0.12.4) (27) with -s reverse was used to obtain feature level raw counts. Raw counts were annotated using package biomart (version 2.42.1 (28), using function:

useEnsembl(biomart=“ensembl”, dataset=“hsapiens_gene_ensembl”, GRCh=38). Entries with no gene symbol were deleted. Then raw counts were normalized and log_2_ transformed using function rlogTransformation from the DESeq2 package [version 1.16.1 (29)]. An increment was added to the normalized values to make all values positive. For identification of differentially expressed genes (DEGs), package LIMMA [version limma_3.42.2 (30, 31)] was used with function model.matrix(~ 0 + group). Volcano plots were generated with the package EnhancedVolcano, version 1.8.0 (32). Pathway analyses of DEGs were performed using the R software package cluster Profiler [version 3.14.3 (33)]. For beeswarm graphs of expression levels, package beeswarm (version 0.2.3) (34). was used. Heatmaps were generated with the function heatmap2 of package gplots (version 3.1.1; https://github.com/talgalili/gplots). VENN diagrams were generated with the function vennPlot (http://faculty.ucr.edu/~tgirke/Documents/R_BioCond/My_R_Scripts/overLapper.R). Deconvolution analysis was performed with the package immunedeconv [version 2.0.4 (35)] and the method mcp_counter (36). We used the R package WGCNA (weighed gene correlation network analysis, version 1.69) for cluster network construction (37) including all genes in the analysis. Hierarchical average linkage clustering was used to construct a dendrogram and identify gene co-expression modules that contain the maximal sets of inter-connected genes. In this study, the following parameters were used for WCGNA: TOMType: unsigned network, minModuleSize: 30, reassignThreshold: 0, power: 10, mergeCutHeight = 0.25.

### Statistical analyses

Analysis and visualization of expression data was performed using the R software package (version 3.4.0) (38). For identification of differentially expressed genes (DEGs), package LIMMA (30, 31) was used with function model.matrix(~ 0 + group) with an adjusted p-value of < 0.05 and an absolute 0.58-fold ([log2] > 1.5) difference in expression levels. For dotplots and cnetplots of pathways, the top 20 pathways, ordered by adjusted p-value as calculated by the package clusterProfiler (33)were selected for presentations in the figures.

## Results

### Description of human cohort

Demographic and clinical characteristics of 88 COVID-19 participants are summarized in **Table 1**. Based on WHO severity levels, participants were divided into 3 groups: mild (WHO severity levels 2-4), moderate (WHO severity level 5) and severe (WHO severity levels 6-9). Gender proportion for the three groups were: 18 (58%) males for the mild group; 18 (72%) males for the moderate group, and 20 (62%) males for the severe group. Median age for the three groups were: 55 years (IQR: 43.0-72.5, range 31-89yr) for the mild group; 69 years (IQR: 56.0-80.0, range 40-89yr) for the moderate group, and 59 years (IQR: 51.5-69.0, range 24-82yr) for the severe group. All subjects across the 3 groups (n=88) were hospitalized. Mean length of hospital stay was 15 days for the mild, 14 days for the moderate and 31 days for the severe group. Four (12%) subjects from the mild group and 5 (20%) subjects from the moderate group were admitted to ICU. Twenty (56%) subjects from the severe group were admitted to ICU with a longer length of stay (mean of 21 days). Mortality rate was higher in the severe group (40%) compared to the mild (32%) and moderate (24%). Seventy-one healthy volunteers were included as healthy controls. Median age of the healthy controls was 50 years (IQR: 44.25-54, range 25-61yr with 50:50 gender ratio).

**Table 1:** Demographics and clinical characteristics of participants.

	HC (n=71)	Mild (2-4) (n=31)	Moderate (5) (n=25)	Severe (6-9) (n=32)	p values_ HC vs mild	p values_ HC vs moderate	p values_ HC vs severe	p values_ mild vs moderate	p values_ mild vs severe	p values_ moderate vs severe
Gender (males/females)	36M/35F	18M/13F	18M/7F	20M/12F	ns	ns	ns	ns	ns	ns
Age/years (median; IQR)	50 (44.25-54)	55 (IQR: 43.0-72.5)	69 (IQR: 56.0-80.0)	59 (IQR: 51.5-69.0)	0.05	<0.0001	<0.05	0.10	>0.99	0.70
day_past_on_sympt		6	8	7	N/A	N/A	N/A	ns	ns	ns
Outcomes										
LOS in hospital (days)		15	14	31	N/A	N/A	N/A	ns	<0.05	<0.0001
Admission to ICU		4 (12%)	5 (20%)	20 (56%)	N/A	N/A	N/A	ns	<0.0001	0.001
LOS in ICU (days)		N/A	7	21	N/A	N/A	N/A	N/A	N/A	<0.05
Death		1 (32%)	6 (24%)	13 (40%)	N/A	N/A	N/A	<0.05	<0.05	Ns

P values were calculated as follows: continuous variables by Kruskal-wallis test, non- parametric, adjusted p value for multiple comparison. Categorial variables by Contingency, chi-square. P value < 0.05 is considered statistically significant. LOS: Length Of Stay; ns: not significant; N/A: not applicable.

### Differentially expressed genes correlate with the levels of severity with WHO severity level 5 representing a transitional state between mild/moderate and severe disease states

**Figure 1:**
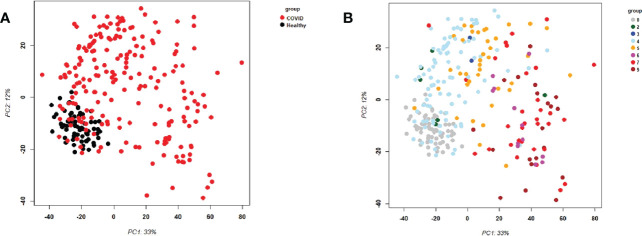
Principal Component Analysis (PCA) of all samples. PCA plot of PC1 and PC2. **(A)** Representing infected participants (COVID) and healthy controls (Healthy) by different colors. **(B)** Representing infected (COVID) participants by WHO severity (levels 2 to 9) and healthy controls (level 0) by different intensities of red. Abbreviations: 0, 2, 3, 4, 5, 6, 7, and 9 stand for WHO severity level 0, 2, 3, 4, 5, 6, 7, and 9, respectively. Note that our cohort did not have participants at levels 1 and 8.

First, we examined the overall variance in sample transcriptomes by principal component analysis (PCA). The PCA showed an excellent separation between healthy controls and infected participants (**Figure 1A**). In addition, the levels of severity showed a clear trend from less to more severe (**Figure 1B**). No obvious separation was observed for sex or sampling site (data not shown).

We then compared the responses at the different severity levels (levels 2-9) to healthy controls (level 0). Only severity levels 0, 4, 5, 7, 9 had reasonably high numbers of samples to perform a contrast to healthy controls (n for level 0: 71, n for level 2: 6, n for level 3: 2; n for level 4: 93; n for level 5: 38; n for level 6: 12; n for level 7: 32; n for level 8: 0; n for level 9: 20). For each severity level against healthy controls, a large number of differentially expressed genes (DEGs) could be identified (**Figure S1A**). The number of DEGs was lowest for severity level 4 (306 up- and 90 down-regulated DEGs), it increased to level 5 (811 up- and 301 down-regulated DEGs), to level 7 (1583 up- and 1089 down-regulated DEGs), to level 9 (1768 up- and 1118 down-regulated DEGs). A strong increase in the number of DEGs was observed between level 5 and lower versus level 7 and higher. The overlaps between the individual comparisons showed that severity level 7 and 9 shared many DEGs (**Figure S1B**). Severity level 5 overlapped to some degree with these two levels, and severity level 4 showed the least number of overlapping DEGs (**Figure S1B**). These results revealed a qualitative and quantitative difference between severity levels 7 and higher compared to severity levels 5 and lower. Complete lists of differentially expressed genes are provided in **Supplementary Datasheets S2**-**S6**.

**Figure 2:**
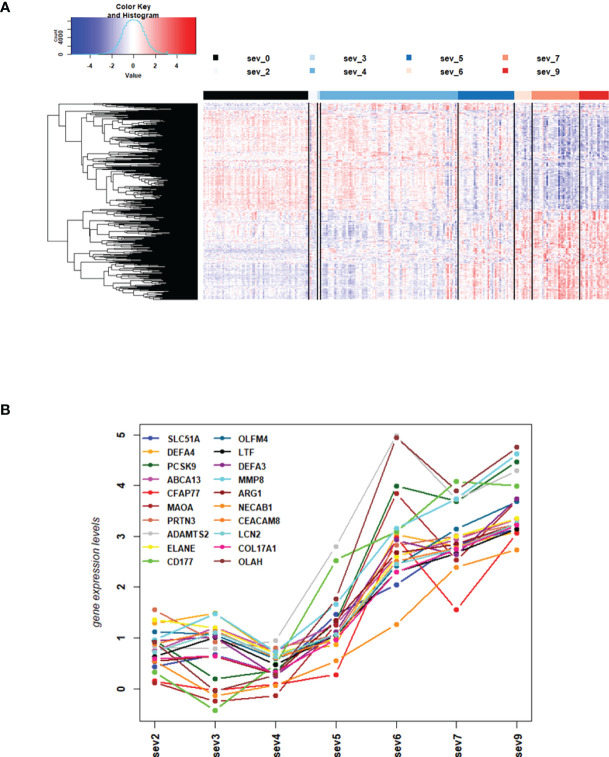
DEGs from comparisons of WHO severity level groups. **(A)** Heatmap of expression levels for top 500 DEGs from the comparison of WHO severity levels 7 + 9 versus 4 + 5. Values were scaled by row. red: up-regulated DEGs, blue: down-regulated DEGS, sev0 to sev9: severity levels 0 to 9, respectively. **(B)** Mean gene expression differences for all severity levels compared to healthy controls for top 20 up-regulated DEGs from the comparison of WHO severity scores 7 + 9 versus 4 + 5. Y-axis: gene expression differences as log fold change, X-axis: severity level groups. Note that our cohort did not have participants at levels 1 and 8. Abbreviations: sev0, 2, 3, 4, 5, 6, 7, and 9 stand for WHO severity level 0, 2, 3, 4, 5, 6, 7, and 9, respectively. These results demonstrate the intermediate (moderate severity) of levels 5 versus mild severity levels (1-4) and severe levels (6-9).

We then contrasted the combined responses from severity level 4 and 5 against the combined responses from severity level 7 and 9 to identify DEGs that were differentially expressed between mild/moderate and severe infections, and we then examined the individual expression levels for all severity levels for the 4 most strongly up- and 4 most strongly down-regulated genes. The corresponding boxplots (**Figures S1C, D**) showed that responses from severity levels 2 to 5 exhibited similar gene expression levels, indicating mild/moderate host responses. However, the expression levels of these genes were strongly up- or down-regulated in severity levels 6 to 9, indicating a strong host response at severity levels 6 and higher. The heatmap for these DEGs (**Figure 2A**) confirmed the conclusions from the 8 DEGs boxplots. It is worth noting that severity level 5 gene expression levels in the heatmap (**Figure 2A**) as well as for the top 20 up-regulated DEGs (**Figure 2B**) were ‘between’ the 1-4 and the gene expression levels of 6-9 severity levels (**Figures 2A, B**) indicating a ‘transitional state’ from mild to severe at this level. Most interestingly, the WHO classification grouped levels 1 to 5 into mild/moderate and 6 to 9 into severe disease categories. Thus, our results from the molecular studies correlate well with these clinical classifications and further relate molecular quantitative measurements to these severity levels.

### Differentially expressed genes correlate with the WHO severity levels and show trends of disease progression

**Figure 3:**
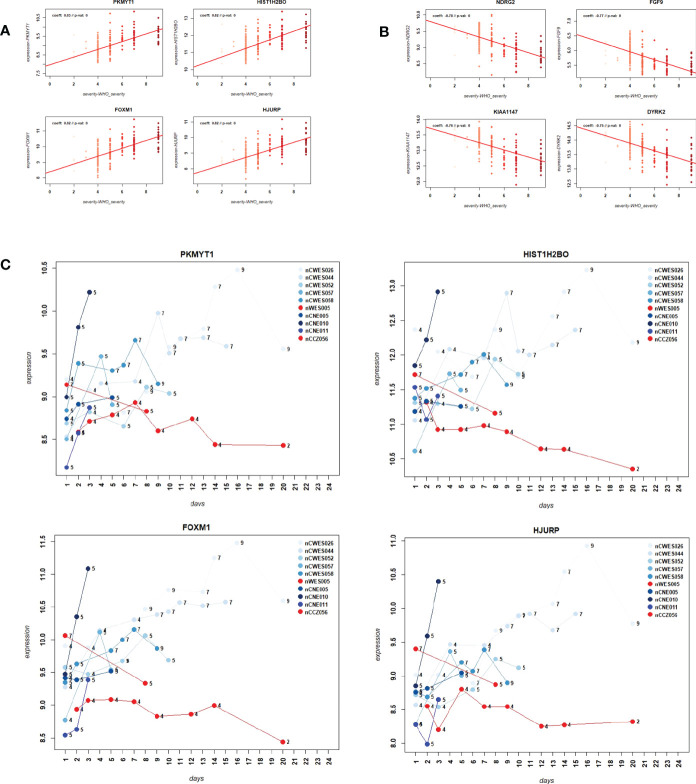
Correlation of gene expression levels with WHO severity levels. **(A)** Scatter plot for the four most strongly positively correlated DEGs with WHO severity levels. Y-axis: gene expression levels for indicated genes, X-axis: WHO severity levels 0 to 9. **(B)** Scatter plot for four most strongly negatively correlated DEGs with severity levels. Y-axis: gene expression levels for indicated genes, X-axis: WHO severity levels 0 to 9. **(C)** Expression levels over time for ten paarticipants for correlated genes *PKMYT1, HIST1H2BO, FOXM1 and HJURP*. Y-axis: gene expression levels for indicated genes, X-axis: day of sample collection after hospital admission of individual patient. Numbers on the line graphs stand for WHO severity levels at the time of sample collection. Note that not all participants were sampled at all the time points.

We then sought to identify genes that were correlated with WHO severity levels. These genes may best represent suitable biomarkers to classify severity levels and potentially predict progression from mild/moderate to severe disease. For this approach, we used the DEGs that were identified in the comparison of severe cases versus healthy controls (see below) and correlated them to severity levels using a linear regression model. Applying a threshold of abs (> 0.5) for the correlation coefficient and a multiple testing adjusted p-value of < 0.05, 1885 genes were significantly correlated with these severity levels. **Figures 3A, B** show the top four genes among the positively or negatively correlated genes, which are *PKMYT1, HIST1H2BO, FOXM1*, and *HJURP*. **Supplementary Datasheet S7** lists all correlated genes. These highly correlated genes showed a clear linear correlation with severity levels and might be suitable as biomarkers. However, we could not perform biomarker classification/prediction with this cohort, because there were too few participants with multiple time points. Nevertheless, we could visualize the changes of expression in these genes for ten selected participants, who displayed different disease trajectories over the period of sample collection. Three of these participants progressed from mild to severe (WHO severity levels 4 to 9), and three progressed from mild to moderate (WHO severity levels 4 to 5). In both groups, there was a positive correlation between the disease scores and expression of the top four positively correlated DEGs (**Figure 3C**). It was also true for two of those participants whose severity score reduced from 7 to 5 or 4 to 2, correlating with the decrease in the gene expression.

### Weight gene correlation network analysis reveals pathways that are associated with the severity levels

**Figure 4:**
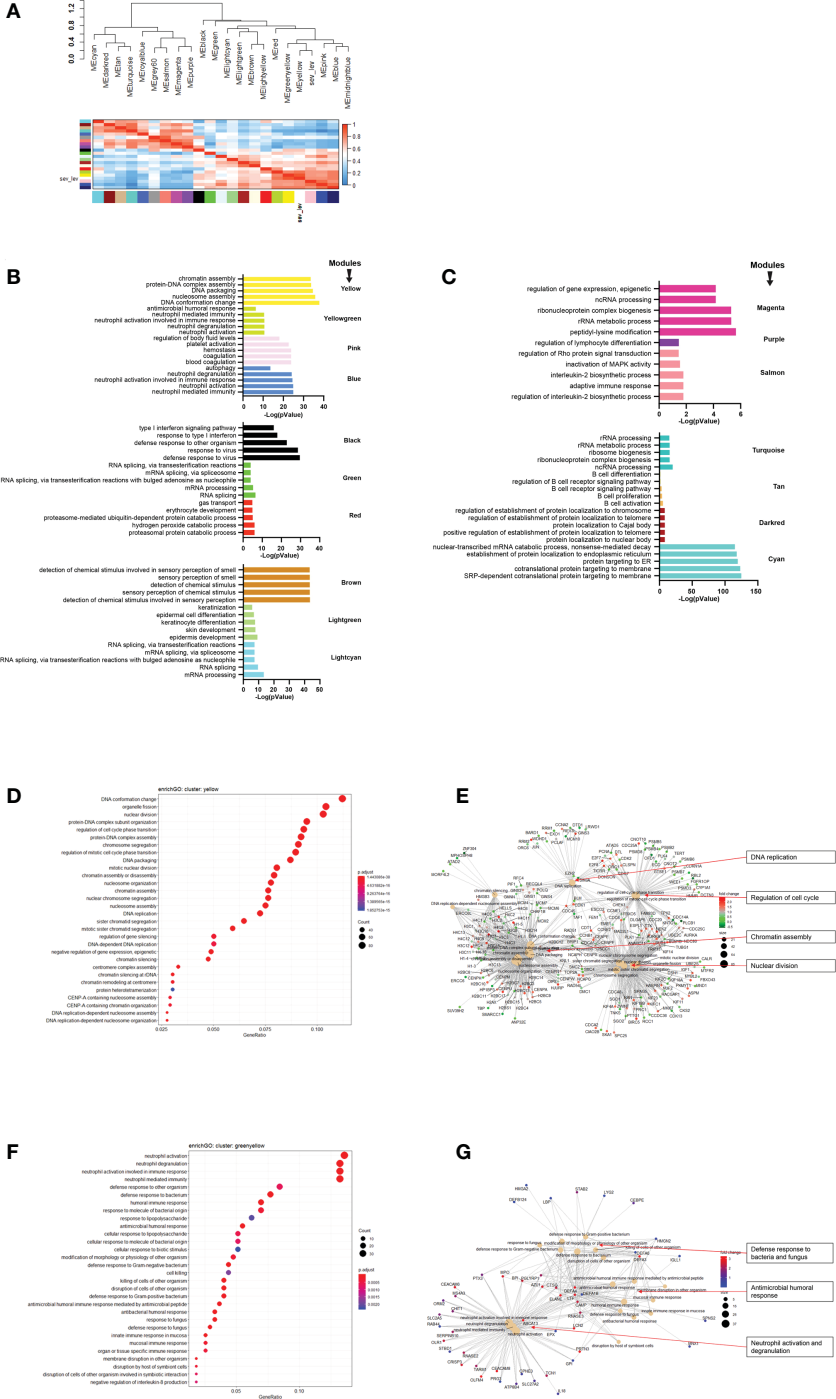
Modules from Weighed Gene Correlation Analysis (WGCNA). **(A)** Dendrogram showing clustering of modules identified by WGCNA. WHO severity levels are correlated with yellow and greenyellow modules. **(B)**& **(C)** Bar diagram showing the top 5 (by adjusted p-value) pathways associated with each of the 17 modules as identified by WGCNA. The significance level is indicated as -Log_10_(adjusted p-value). **(D)** Functional analysis using GO term enrichment for genes in yellow module showing the 30 most significant pathway annotations. **(E)** cnetplot illustrating relationship of genes from yellow module to pathways. **(F)** Functional analysis using GO term enrichment for genes in greenyellow module showing the 30 most significant pathway annotations. **(G)** cnetplot illustrating relationship of genes from greenyellow cluster to pathways. Nodes in cnetplots represent pathways significantly associated with the genes from the respective module. Genes from the module are connected to these nodes with color-coded log-fold changes from the contrast between severe cases versus healthy controls. Abbreviations: sev_lev stands for WHO severity levels.

Next, we performed WGCNA which applies an unsupervised clustering method to group genes into modules of co-regulated genes. This analysis revealed 21 modules, which were grouped into two main branches (**Figure 4A**). The top five pathways associated with each module (with adjusted p-vale < 0.05), are shown in **Figures 4B, C**. Clusters greenyellow (neutrophil activation and degranulation, antimicrobial humoral response, **Figures 4D, E**) and yellow (cell cycle and replication, **Figures 4F, G**) grouped together with severity levels in the dendrogram (**Figure 4A**). For 17 modules, we identified associated GO term enrichment pathways (**Figures S2A–V**). An overview of these clusters with number of genes, major GO pathway annotations and significant correlation to severity categories are listed in **Supplementary Datasheet S8**.

### Differentially expressed genes reveal strong differences in molecular pathway regulation related to mild/moderate and severe disease categories

**Figure 5:**
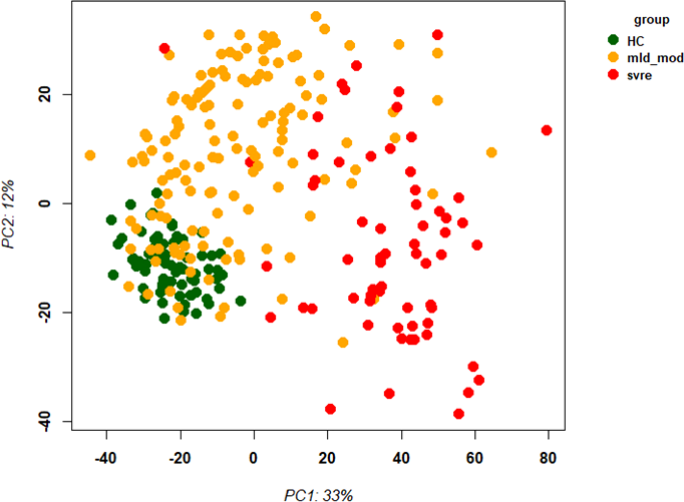
Principal Component Analysis (PCA) of all samples. PCA plot of PC1 and PC2 representing different severity categories (mild, moderate, severe, and healthy) by colors. Abbreviations: HC stands for healthy controls; mld_mod stands for mild and moderate; svre stands for severe.

**Figure 6:**
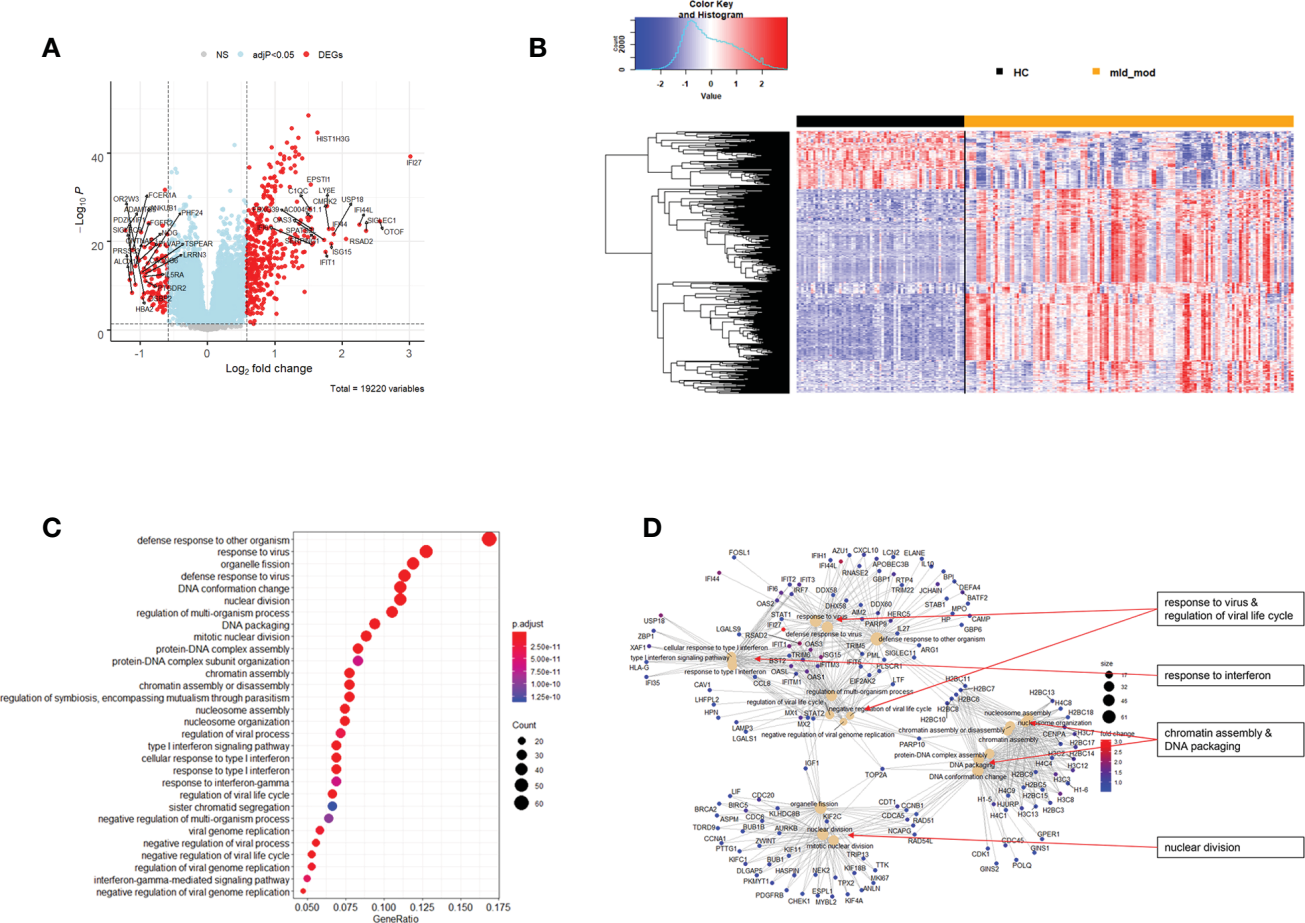
Differential genes expression analysis contrasting mild/moderate cases to healthy controls. **(A)** Volcano plot of results of the contrast from the linear regression analysis. y-axis: -log_10_ BH multiple testing adjusted p-values, x-axis: log_2_ fold change. DEGs (absolute log-fold change > 1.5, corresponding to a log_2_-fold change > 0.58; multiple testing adjusted p-value < 0.05) are colored red and the top 20 up- and down-regulated (by log-fold change) DEGs are labeled. Blue: genes with adjusted p-value < 0.05. **(B)** Heatmap of expression levels for DEGs. Values were scaled by row. red: up-regulated DEGs, blue: down-regulated DEGS. HC: healthy controls, mld_mod: mild/moderate pateints. **(C)** Functional analysis using GO term enrichment for up-regulated DEGs showing 30 most significant pathway annotations. No significant pathways could be identified for the down-regulated genes **(D)**: cnetplot illustrating relationship of DEGs to pathway annotations. Nodes in cnetplots represent pathways significantly associated with the differentially expressed genes. The differentially expressed genes are connected to these nodes with color-coded log-fold changes from the contrast between mild/moderate cases versus healthy controls. Abbreviations: HC stands for healthy controls; mld_mod stands for mild and moderate.

**Figure 7:**
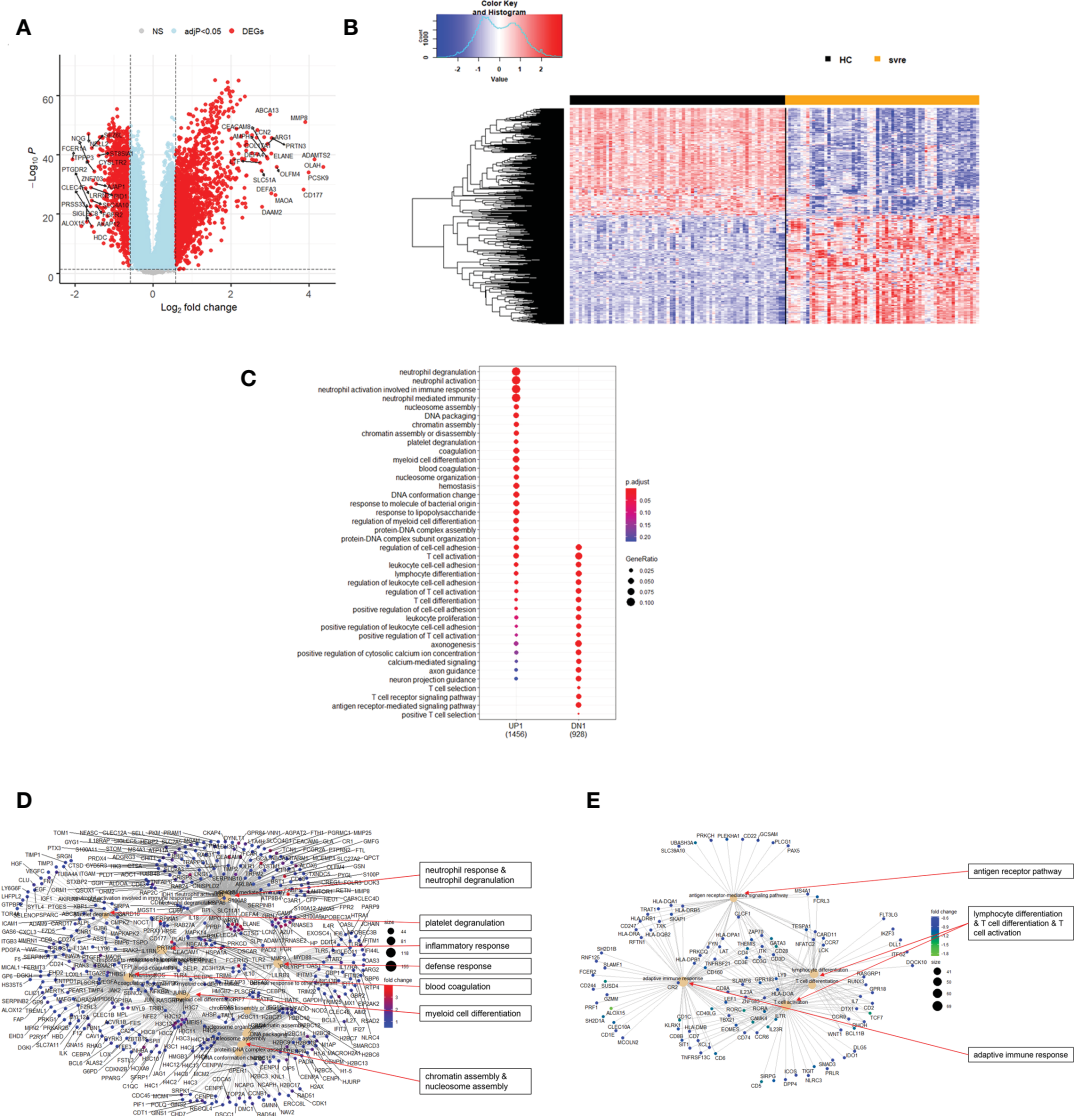
Differential genes expression analysis contrasting severe cases to healthy controls. **(A)** Volcano plot of results of the contrast from the linear regression analysis. y-axis: -log_10_ BH multiple testing adjusted p-values, x-axis: log_2_ fold change. DEGs (absolute log-fold change > 1.5, corresponding to a log_2_-fold change > 0.58; multiple testing adjusted p-value < 0.05) are colored red and the top 20 up- and down-regulated (by log-fold change) DEGs are labeled. Blue: genes with adjusted p-value < 0.05. **(B)** Heatmap of expression levels of top 500 (by log fold change) regulated DEGs. Values were scaled by row. red: up-regulated DEGs, blue: down-regulated DEGS. HC: healthy controls, mld/mod: mild/moderate pateints. **(C)** Functional analysis using GO term enrichment for up- and down-regulated DEGs showing 20 most significant pathway annotations for both groups. **(D)** cnetplot illustrating relationship of up-regulated DEGs to pathway annotations. **(E)** cnetplot illustrating relationship of down-regulated DEGs to pathway annotations. Nodes in cnetplots represent pathways significantly associated with the differentially expressed genes. The differentially expressed genes are connected to these nodes with color-coded log-fold changes with color-coded log-fold changes from the contrast between severe cases versus healthy controls. Abbreviations: HC stands for healthy controls; svre stands for severe.

**Figure 8:**
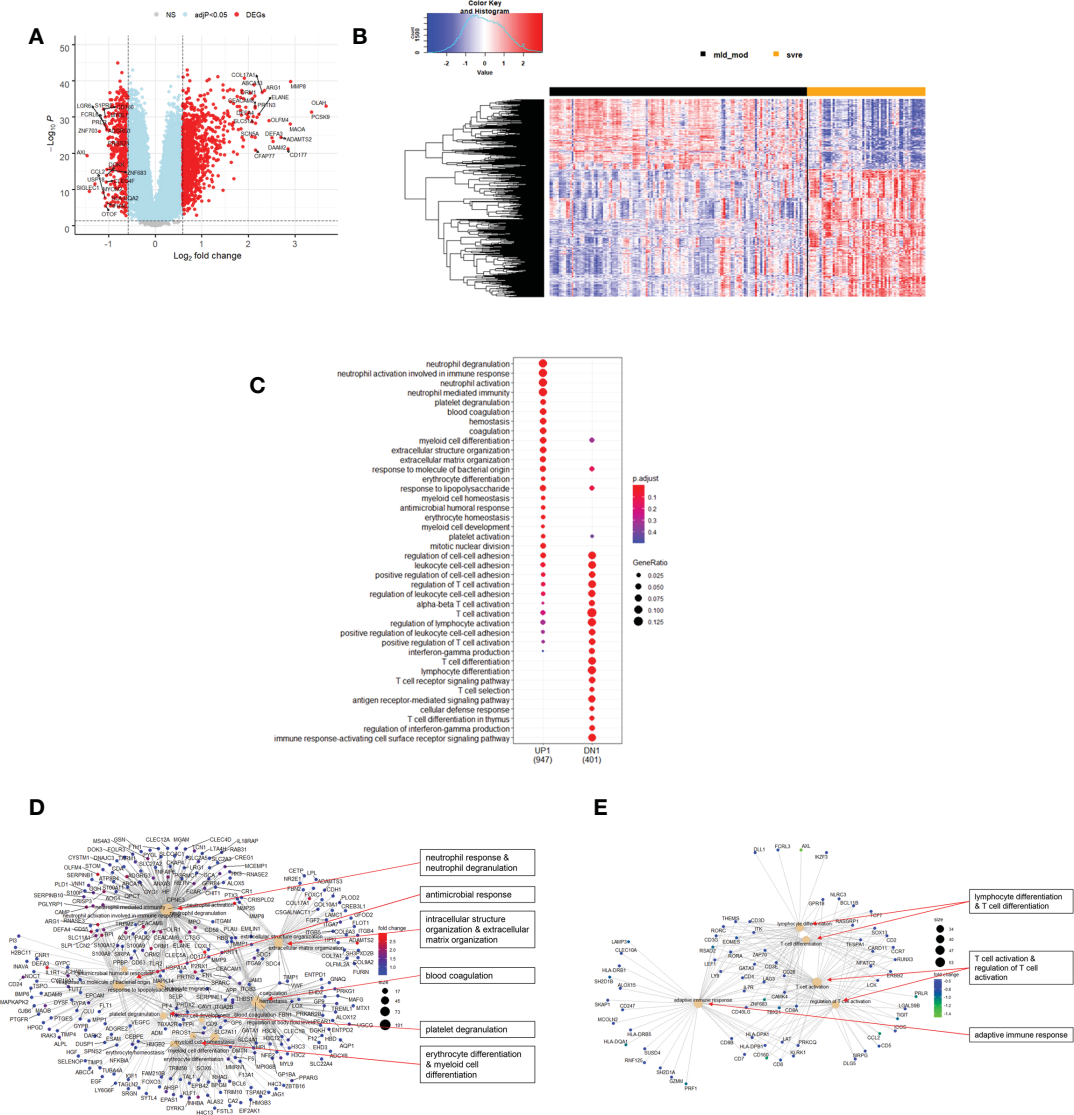
Differential genes expression analysis contrasting mild/moderate to severe cases. **(A)** Volcano plot of results of the contrast from the linear regression analysis. Y-axis: -log_10_ BH multiple testing adjusted p-values, x-axis: log_2_ fold change. DEGs (absolute log-fold change > 1.5, corresponding to a log_2_-fold change > 0.58; multiple testing adjusted p-value < 0.05) are colored red and the top 20 up- and down-regulated (by log-fold change) DEGs are labeled. Blue: genes with adjusted p-value < 0.05. **(B)** Heatmap of expression levels of top 500 (by log fold change) regulated DEGs. Values were scaled by row. Red: up-regulated DEGs, blue: down-regulated DEGS. HC: healthy controls, mld/mod: mild/moderate pateints. **(C)** Functional analysis using GO term enrichment for up- and down-regulated DEGs showing 20 most significant pathway annotations for both groups. **(D)** cnetplot illustrating relationship of up-regulated DEGs to pathway annotations. **(E)** cnetplot illustrating relationship of down-regulated DEGs to pathway annotations. Nodes in cnetplots represent pathways significantly associated with the differentially expressed genes. The differentially expressed genes are connected to these nodes with color-coded log-fold changes from the contrast between severe versus mild/moderate cases. Abbreviations: mld_mod stands for mild and moderate; svre stands for severe.

The above analyses of WHO severity levels revealed two main severity categories: mild/moderate including WHO severity levels 1-5 and severe including WHO severity levels 6-9. **Figure 5** demonstrates clear separation of these groups in a PCA. Using these newly defined severity categories, we then performed differential gene expression (DEG) analyses between these groups and the healthy controls. The comparison of the healthy controls to the mild/moderate cases revealed 487 DEGs (380 up- and 107 down-regulated genes, **Figures 6A, B**)) and between the healthy controls and the severe cases identified 2580 DEGs (1558 up- and 1022 down-regulated genes, **Figures 7A, B**). The comparison of the mild/moderate cases to the severe cases identified 1448 DEGs (1013 up- and 435 down-regulated genes, **Figures 8A, B**). Thus, the severe cases exhibited a much stronger change in gene expression in the peripheral blood compared to the healthy controls than did the mild/moderate cases. Furthermore, there was a large overlap between the comparison of the healthy controls with the severe cases and the comparison of the mild/moderate cases to the severe cases (data not shown). This observation indicates a strong jump of the host response in the peripheral blood from the mild/moderate to the severe cases. Complete lists of differentially expressed genes are provided in **Supplementary Datasheets S9**-**S11**.

Functional analysis of the up-regulated DEGs from the comparison of the mild/moderate cases versus the healthy controls identified pathways associated with host response to viral infections and activation of interferon responses (**Figures 6C, D**; **Table 2**). No pathway association was found for the down-regulated genes. Analysis of the up-regulated DEGs from the comparison of the severe cases versus the healthy controls identified pathways associated with inflammatory and innate immune cell responses and pathways related to blood coagulation and neutrophil degranulation (**Figures 7C–E**; **Table 2**). The latter responses have been described as the hallmark clinical symptoms in severe COVID-19 participants. Pathway associations for the DEGs from the comparison of the severe versus the mild/moderate cases were similar to the comparison of the severe cases versus the healthy controls (**Figures 8C–E**; **Table 2**).

**Table 2:** Pathway associations of DEGs regulated in different severity categories.

Infection	Direction	Figure	Associated Pathways
Mild/moderate versus healthy controls	UP	6C, D	• response to virus & regulation of viral life cycle • response to interferon • chromatin assembly & DNA packaging • nuclear division
Mild/moderate versus healthy controls	DOWN	NA	• None
Severe versus healthy controls	UP	7C, D	• neutrophil response & neutrophil degranulation • platelet degranulation • inflammatory response • defense response • blood coagulation • myeloid cell differentiation • chromatin assembly & nucleosome assembly
Severe versus healthy controls	DOWN	7C, E	• antigen receptor pathway • lymphocyte differentiation & T cell differentiation & T cell activation • adaptive immune response
Severe versus Mild/moderate	UP	8C, D	• neutrophil response & neutrophil degranulation • antimicrobial response • intracellular structure organization & extracellular matrix organization • blood coagulation • platelet degranulation • erythrocyte differentiation & myeloid cell differentiation
Severe versus Mild/moderate	DOWN	8C, E	• lymphocyte differentiation & T cell differentiation • T cell activation & regulation of T cell activation • adaptive immune response

NA, Not Applicable.

We then correlated all WGCNA modules with severity categories using ANOVA. Applying an adjusted p-value of 0.01, we identified 20 modules that were significantly correlated with severity categories using their eigengene values for each patient (**Supplementary Datasheet S12**). Gene expression levels from the eigengenes of 14 modules were significantly different between severe and moderate cases, and also between severe cases and healthy controls (**Supplementary Datasheet S12**). Modules blue (**Figure S2B**) and greenyellow (**Figure S2L**) included neutrophil activation pathways, the pink module included platelet responses and coagulation (**Figure S2H**), and the salmon module (**Figure S2N**) included adaptive immune response pathways. The cluster memberships and eigengene values are listed in **Supplementary Datasheet S13**.

### Deconvolution analysis reveals changes in the immune cell composition associated with the severity categories

**Figure 9:**
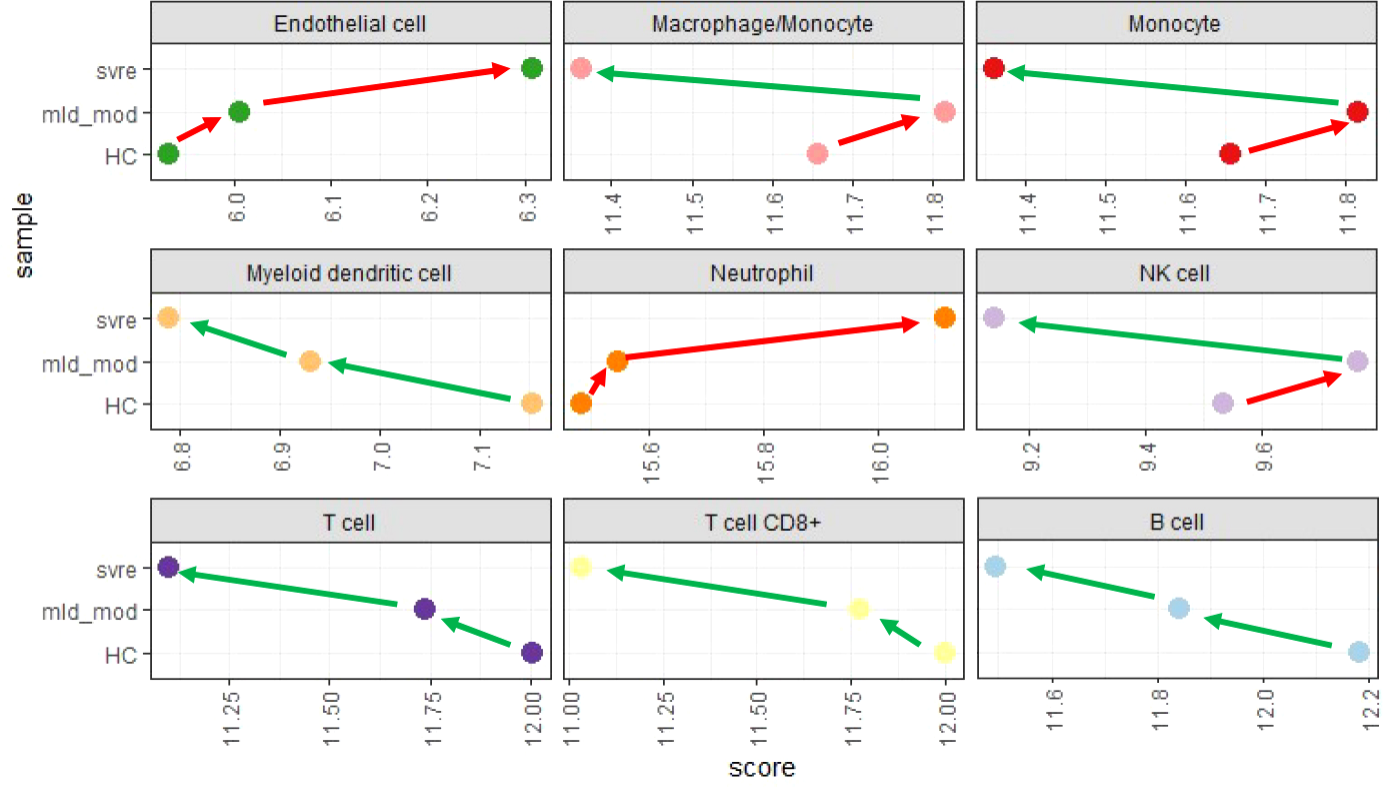
Deconvolution analysis. Mean values for mild/moderate, severe and healthy control groups were calculated and subjected to deconvolution analysis. Y-axis: severity categories, X-axis: scores from mcp_counter analysis for the different cell populations. Abbreviations: HC stands for healthy controls; mld_mod stands for mild and moderate; svre stands for severe.

We then performed a deconvolution analysis, which uses known cell-type specific gene sets to estimate the relative abundance of different immune cell subpopulations. This deconvolution analysis revealed a slight increase in endothelial cell and neutrophil in the mild/moderate cases compared to the healthy controls. However, a much stronger increase was observed in the severe cases (**Figure 9**). Conversely, for B, T, and dendritic cells, a slight decrease was observed for the mild/moderate cases compared to healthy controls whereas a strong decrease was evident in the severe cases compared to either healthy controls or mild/moderate cases (**Figure 9**). For macrophage/monocyte and NK cells, an increase in their relative abundance was observed in the mild/moderate cases compared to the healthy controls. There was a significant reduction in the severe cases compared to both the health controls and the mild/moderate cases (**Figure 9**).

## Discussion

Current classification of COVID-19 severity by WHO places patients under 3 broad categories (mild, moderate, and severe) and each category is subdivided into 2-3 severity levels based on location and supportive measures, e.g., mild = levels 1-3, moderate = levels 4-5, and severe = 6-9 (2). Each level, though under the same category, represents a different level of medical support. For example, under the “moderate” category, severity level 5 requires oxygen by mask or nasal prongs whereas level 4 does not require any oxygen therapy. Most of the previous studies have compared host response between patients from different WHO severity categories or with different clinical symptoms (6, 13, 18), yet the differences among individual levels of the severity are not well understood. Our current study was designed to address this gap and aimed to provide deeper understanding of the role of each severity level during disease progression by including information on changes and differences in whole blood gene expression profiles.

Here, we analyzed the transcriptomic signature associated with severity levels 0, 2-7, and 9 and observed 4 distinct groups based on similarity in their transcriptomes: 1) level 0; 2) levels 2-4; 3) level 5; 4) levels 6/7/9. Overall, our grouping based on transcriptomics had a very good match with WHO categories for the uninfected (level 0) and the severe (5–8) categories. However, for the “mild” and “moderate” cases, we observed some discordance between our grouping and WHO severity category. Data-driven re-grouping of COVID-19 patients has also been observed previously in another study by Aschenbrenner et al. (13). Our data showed that severity level 5 did not group with level 4 though both levels are classified as “moderate” under WHO category. The transcriptome of severity level 5 stands out as a transitional stage between the levels 2-4 and the levels 6/7/9. This finding not reported elsewhere, correlates with the different levels of medical support as observed for level 4 and 5, i.e., oxygen support is required for level 5 but not level 4. Hence transcriptomic grouping may better predict or discern disease progression compared to one solely based only on clinical manifestation. To explore further, we looked at the expression of four genes over time in ten participants who displayed very different disease courses: 6 with progressive severity (WHO 4 to 9 or WHO 4-5), and 2 with regressive severity (WHO 7 to 5 or WHO 4 to 2), and 2 with unchanged severity (WHO 5). *PKMYT1, HIST1H2B0, FOXM1, HJURP* are the top four genes among those that positively correlated with severity levels. Our data showed that for the participants with either progressive or regressive severity over time, expression profile of these four genes fit well with the changes of disease severity levels. However, in the two participants with unchanged severity at WHO 5, expression profile of these four genes presented a rather concerning disease progression which however had not been reflected in the clinical manifestation. Though we cannot not draw any general conclusion based on just ten participants, our data did demonstrate the need of future prospective studies to validate these genes in predicting disease progression. A particular focus on the host response in the moderate group (WHO 5) could lead to potential therapeutic targets forcing a regressive rather than a progressive course.

Currently, host response biomarkers used for predicting COVID-19 severity include proinflammatory cytokines (IL6, TNF, IL8, etc.), inflammatory markers (CRP, procalcitonin, and ferritin, etc.), neutrophil/lymphocyte ratio, and lymphopenia (39). These biomarkers are useful indicators of severe COVID-19 but do not have the power to discern patients who are yet to develop severe disease. Genes identified in our data, showed strong correlation with disease severity levels, and would contribute towards discovery of novel genomic biomarkers that would help address the issues with currently available biomarkers.

To gain further insight into the pathways and cellular processes that are crucial for regulating disease outcome, we performed WGCNA analysis to identify modules co-regulated with disease severity. Among the 21 modules identified, the yellow and greenyellow clusters were the closest neighbors of the severity levels. Yellow module is consisted of pathways involved in cell cycle regulation and chromatin assembly/disassembly. Manipulation of host cell cycle has been commonly observed in many types of viruses including SARS-CoV-1 as a mechanism to facilitate their own replication. Virus do so by inducing cell cycle arrest at a certain phase by inhibiting the activation of cyclins and cyclin-dependent kinases (CDKs), the major regulators of mammalian cell cycle (40–43). Our data suggests that SARS-CoV-2 is also able to regulate host cell cycle through similar mechanism. The identification of chromatin assembly/disassembly in the yellow module indicates possible epigenetic alterations of the host genome induced by SARS-CoV-2 infection as it is known that epigenetic modification such as histone acetylation can regulate gene expression *via* chromatin remodeling (44). It has been reported that expression of angiotensin-converting enzyme 2 (ACE2) gene, encoding the membrane receptor essential for viral entry, is regulated by histone modifications and its expression is upregulated in the lung of severe COVID-19 patients (45–47). Our data suggests that epigenetic alteration of host genome in the blood in response to SARS-CoV-2 infection also plays a role in regulating disease severity. The greenyellow module consists of pathways involved in neutrophil activation/degranulation and immune response towards bacteria/fungus. Aberrant activation and enrichment of neutrophil has been well studied as one of the hallmarks for severe COVID-19 (11–13) and our data also supported this observation. The first line of host response towards bacteria/fungus or other pathogens is mediated by toll-like receptors (TLRs), which induce downstream signaling pathways for induction of proinflammatory cytokine production, immune cell activation and interferon production (48). It has been shown that TLR4, one of the TLRs important for recognizing bacterial or fungal pathogens, also recognizes the spike glycoprotein of SARS-CoV-2 leading to increased ACE2 expression and overproduction of inflammatory cytokines (49, 50). Overproduction of inflammatory cytokines (so-called cytokine storm) is observed in the severe COVID-19 patients, as evidenced by elevated serum levels of proinflammatory cytokines (in particularly, interleukin-6 (IL6) and tumor necrosis factor alpha (TNF)), and C-reactive protein (CRP) (3–5). Hence, our WGCNA analyses highlight the important role of cell cycle manipulation, epigenetic regulation of host genome by SARS-CoV-2 infection, neutrophil activation, and innate immune response *via* TLRs, in determining the disease severity.

Transcriptomic signatures associated with COVID-19 severity as identified in the current study corroborate findings from previous studies and a recently published multi-omic blood atlas (51). Firstly, our deconvolution analyses revealed the hallmark lymphopenia (14–16, 51) and neutrophil enrichment (11–13, 51) in the severe group (WHO 6/7/9) compared to mild/moderate group (WHO 2-5). Secondly, genes involved in neutrophil activation were highly upregulated in the severe group compared to HC, including *CD177, MMP8, ELANE, OLFM4*, and *MPO*, which have also been identified in another study (13). WGCNA analyses also confirmed that pathways involved in neutrophil activation/degranulation, together with antimicrobial response and activation of blood coagulation, were significantly correlated with COVID-19 severity, in keeping with findings from the blood atlas paper (51). Thirdly, IFN-I responses were found to be enriched in the less severe COVID-19 cases, consistent with previous findings (6, 51).

### Limitations of the study

We are aware of some limitations of the current study. All healthy controls (WHO level 0) were recruited from a single center in Australia, whereas the COVID-19 participants were recruited from multiple sites across Australia and Czech Republic. However, our PCA did not indicate any site-specific differences. Furthermore, multiple samples were collected from some participants whereas for most participants, only a single sample was collected. These differences in collection sites and number of data points per patient could potentially create confounding bias in our findings. The fact that our data reproduces many findings from previous studies gives us confidence to assume that there is no strong confounder related to either the collection sites or number of data points per patient. The second limitation of the current study is that we could not perform correlation analyses between the transcriptome of each WHO severity level with clinical inflammatory biomarkers (e.g., CRP, procalcitonin, ferritin, neutrophil/lymphocyte ratio, and lymphopenia, etc.) or clinical severity scores (e.g., APACHE II or SOFA scores). This was due to limited availability of the clinical data. Future prospective studies would be beneficial to assess the correlation between the clinical inflammatory biomarkers or clinical severity scores with the transcriptomic markers as identified in this study. The third limitation is that we were not able to keep the sampling time constant due to the different availability of the staff and the participants, which might have some unforeseen impact on the number and function of the blood cells. The fourth limitation is that few participants of our study cohort were undergoing immunosuppressive therapy at the time of sample collection, however most of our participants were not. Due to our study design, we were not able to assess the impact of the immunosuppressive agents on the blood transcriptomes. A future prospective study would be necessary to investigate this impact. Finally, we do not have molecular identification of the SARS-CoV2 variants infecting the individual participant. Since most samples were collected before 2021, we assume that most were derived from the pre-Delta period.

Overall, our data demonstrated the presence of a transitional level between the mild/moderate and the severe groups. Genes and pathways associated with disease severity, which could potentially be used as prognostic biomarkers or therapeutic targets, were identified.

## Data availability statement

Raw FASTQ data discussed in this publication have been deposited in NCBI's Sequence Read Archive under BioProject accession PRJNA901461. Count data were deposited to NCBI’s Gene Expression Omnibus (52) and are accessible through GEO Series accession number GSExxxxx (https://www.ncbi.nlm.nih.gov/geo/query/acc.cgi?acc=GSE217948). Details can be found in **Supplementary Datasheet S1**.

## Ethics statement

The studies involving human participants were reviewed and approved by Nepean Blue Mountain Local Health District Research Ethics Committee; Western Sydney Local Health District Human Research Ethics Committee. The patients/participants provided their written informed consent to participate in this study.

## Author contributions

Study concept and design: BT and MS, ethics/governance application: BT, MS, AM, YW, and ST, recruitment of participants, sample collection/processing: MS, ST, KK, TP, TK, and BK, clinical data collection and REDCap database: BT, AM, TP, ST, TK, BK, and AP, RNASeq data pre-processing and QC: TC; data analyses: KS, data interpretation and discussion: KS, YW, TC, MS, BT, AM, KS, JI, IT, JA, SM, and JB, manuscript writing and generating figures: KS and YW, manuscript revision: KS, YW, TC, and MS, funding acquisition and project supervision: BT, MS, KS, and AM. All authors contributed to the article and approved the submitted version. PREDICT-19 Consortium contributed to many aspects of this study including study concept and design, applications of material transfer agreements, recruitment of participants, sample collections, clinical data collection, setup of REDCap database, data interpretation and discussions. Members of PREDICT-19 consortium are listed below in alphabetic order according to their first names:

*Alberto Ballestrero, Allan Cripps, Amanda Cox, Amy L Phu, Andrea De Maria, Anthony McLean, Arutha Kulasinghe, Ben Marais, Benjamin Tang, Carl Feng, Damien Chaussabel, Darawan Rinchai, Davide Bedognetti, Gabriele Zoppoli, Gunawan Gunawan, Irani Thevarajan, Jennifer Audsley, John-Sebastian Eden, Jonathan Iredell, Karan Kim, Kirsty Renfree Short, Klaus Schughart, Mandira Chakraborty, Marcela Kralovcova, Marek Nalos, Marko Radic, Martin Matejovic, Maryam Shojaei, Meagan Carney, Michele Bedognetti, Miroslav Prucha, Mohammed Toufiq, Nandan Deshpande, Narasaraju Teluguakula, Nicholas West, Paolo Cremonesi, Philip Britton, Ricardo Garcia Branco, Rodolphe Thiebaut, Rostyslav Bilyy, Sally Teoh, Stephen MacDonald, Tania Sorrell, Thomas Karvunidis, Tiana Maria Pelaia, Tim Kwan, Tracy Chew, Tri Giang Phan, Velma Herwanto, Win Sen Kuan, Ya Wang, and Yoann Zerbib.*

## Supplementary material

Supplementary Figure 1 DEGs from individual severity levels to healthy controls.

Supplementary Figure 2 Pathway analyses of all WGCNA modules.

Supplementary Data Sheet 1 SampleInformation_161122.

Supplementary Data Sheet 2 limma_Sev4vsHC_170622.txt.

Supplementary Data Sheet 3 limma_Sev5vsHC_170622.txt.

Supplementary Data Sheet 4 limma_Sev7vsHC_170622.txt.

Supplementary Data Sheet 5 limma_Sev9vsHC_170622.txt.

Supplementary Data Sheet 6 limma_Sev79vsSev45_170622.txt.

Supplementary Data Sheet 7 correlation_sevLevel_190722_2.txt.

Supplementary Data Sheet 8 Description_WGCNA_cluster_240822.xlsx.

Supplementary Data Sheet 9 limma_modmldvsHC_200622.txt.

Supplementary Data Sheet 10 limma_svrevsHC_200622.txt.

Supplementary Data Sheet 11 limma_svreVSmodmld2_200622.txt.

Supplementary Data Sheet 12 ANOVA_sevCAT_WGCNA_cluster_230622.txt.

Supplementary Data Sheet 13 WGCNA_geneInfo_220622.txt.
