# Supplementary material for: Blood transcriptome responses in patients correlate with severity of COVID-19 disease
Source: Front Immunol. 2023 Jan 20;13:1043219. doi: 10.3389/fimmu.2022.1043219 (PMC9896980; doi:10.3389/fimmu.2022.1043219)

**Supplements**

## Supplement Figure S1: DEGs from individual severity levels to healthy controls

### **(A)** Number of differentially expressed genes for contrasting individual severity levels to healthy controls.


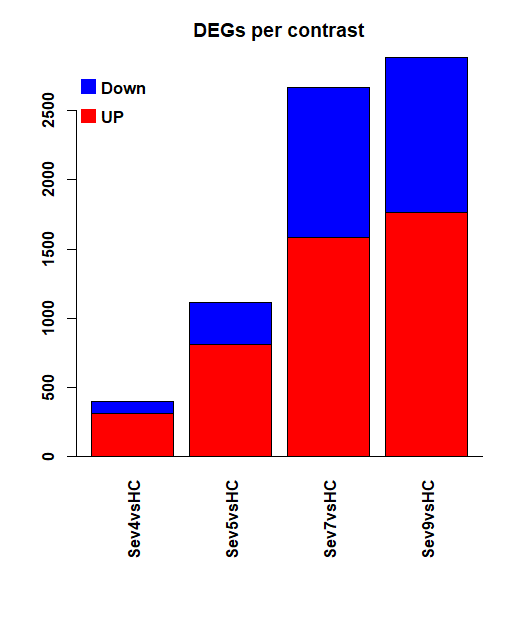


### **(B):** Overlap of DEGs from contrasting individual severity levels to healthy controls


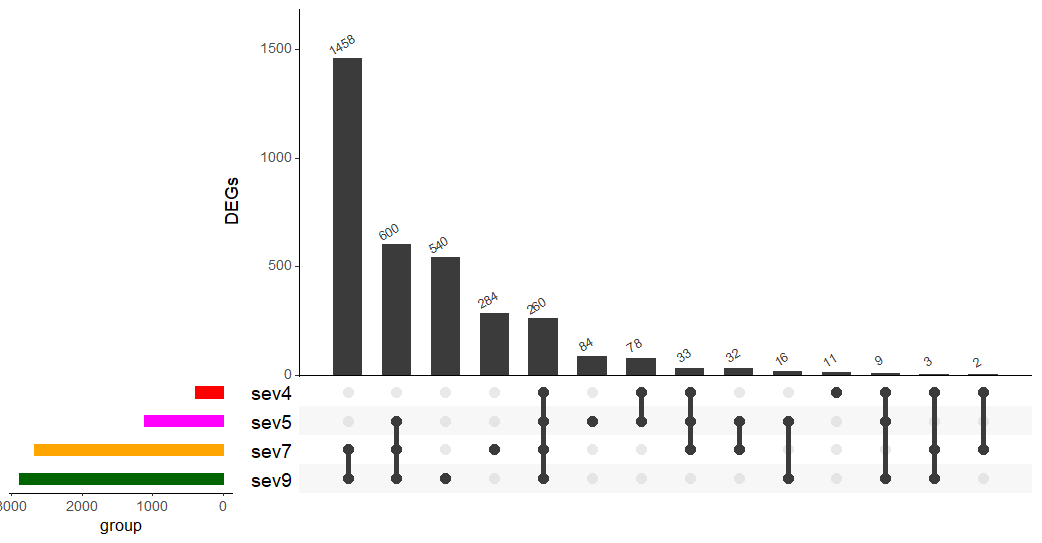


### **(C):** Boxplot of expression levels of the four most strongly up-regulated genes for contrasts of severity levels 4/5 to levels 7/9


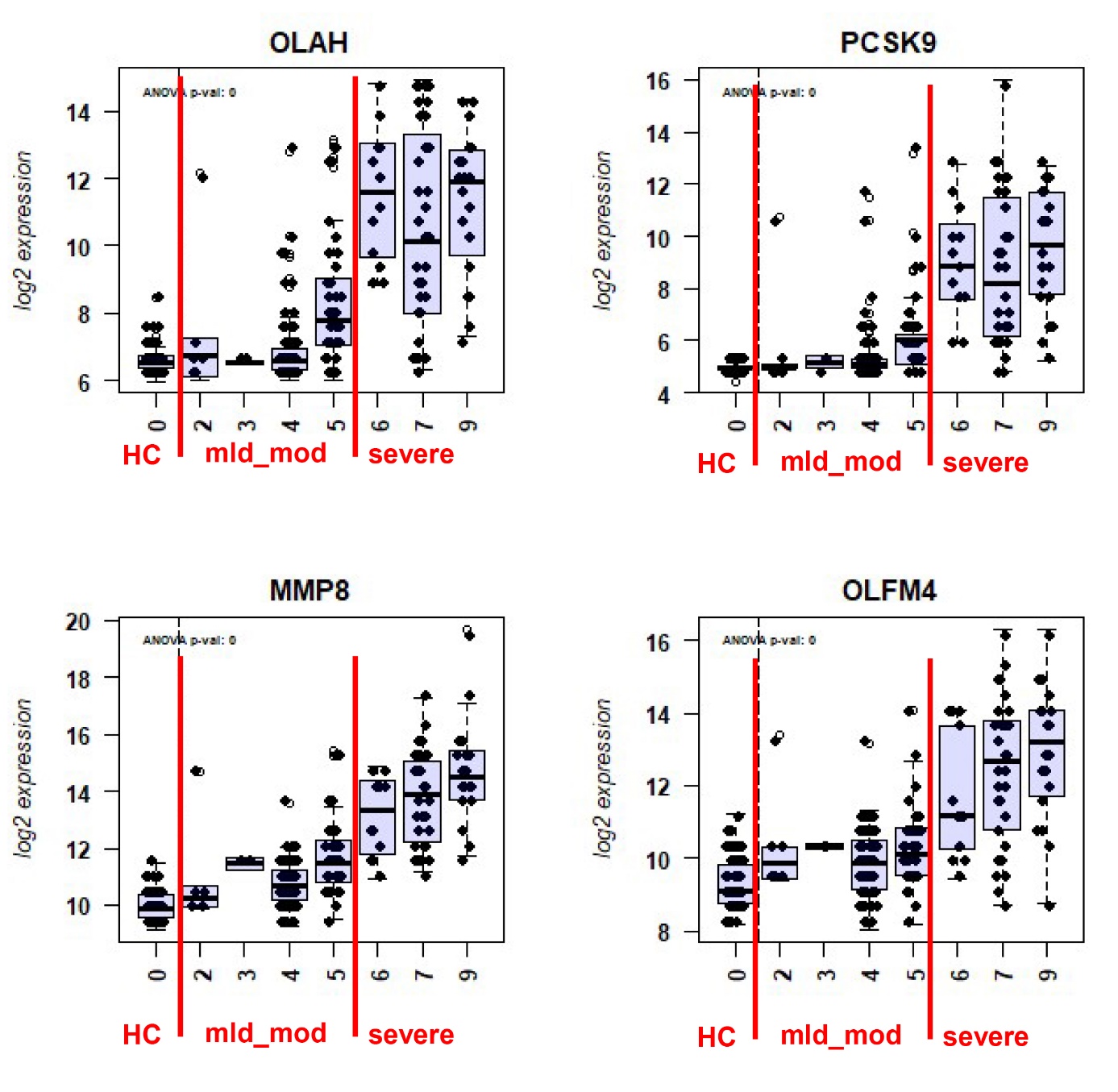


### **(D):** Boxplot of expression levels of the four most strongly down-regulated genes for contrasts of severity levels 4/5 to levels 7/9


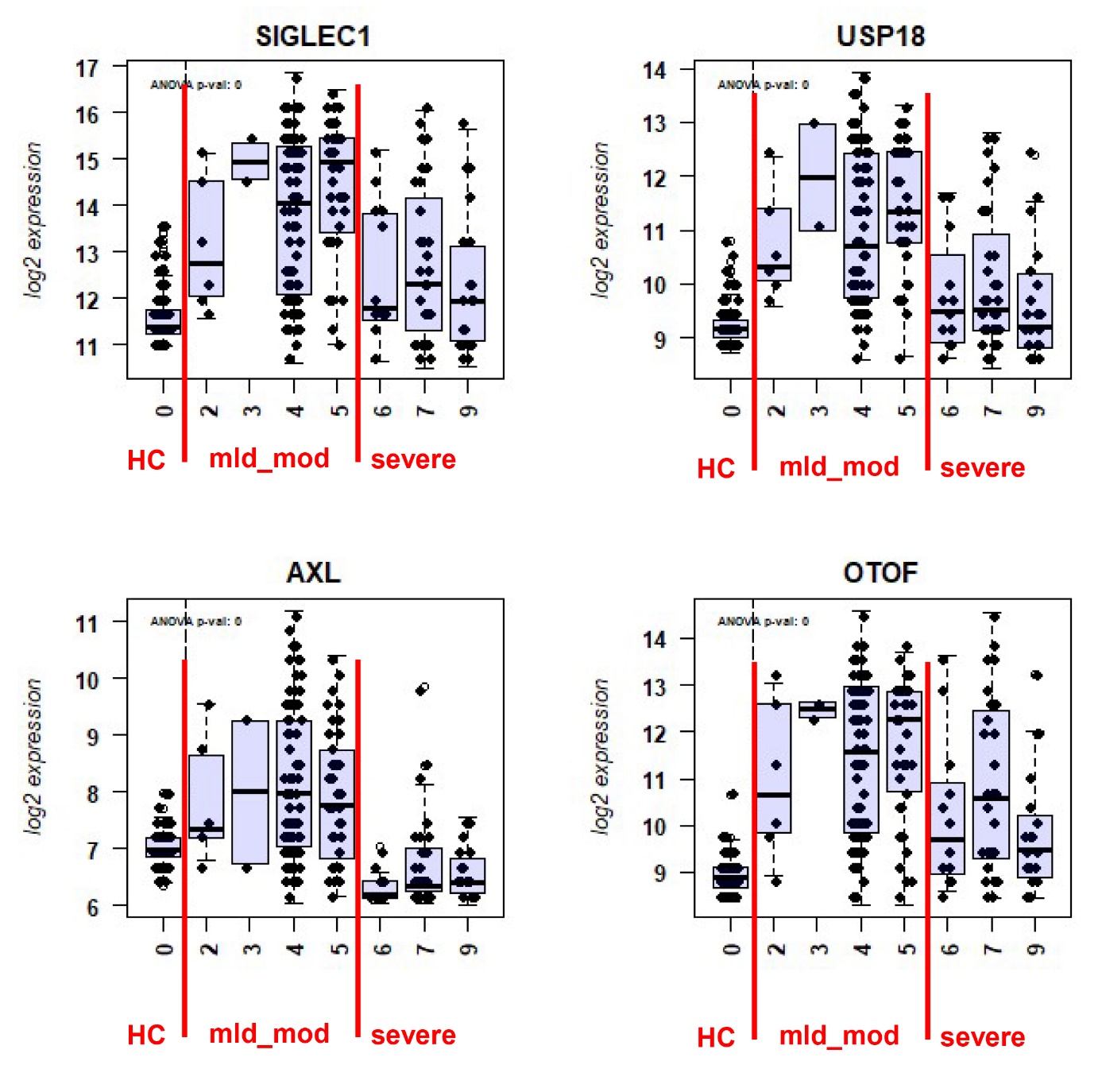


## Supplement Figure S2: Pathway analyses of all WGCNA cluster

### **A: Cluster turquoise - RNA processing**


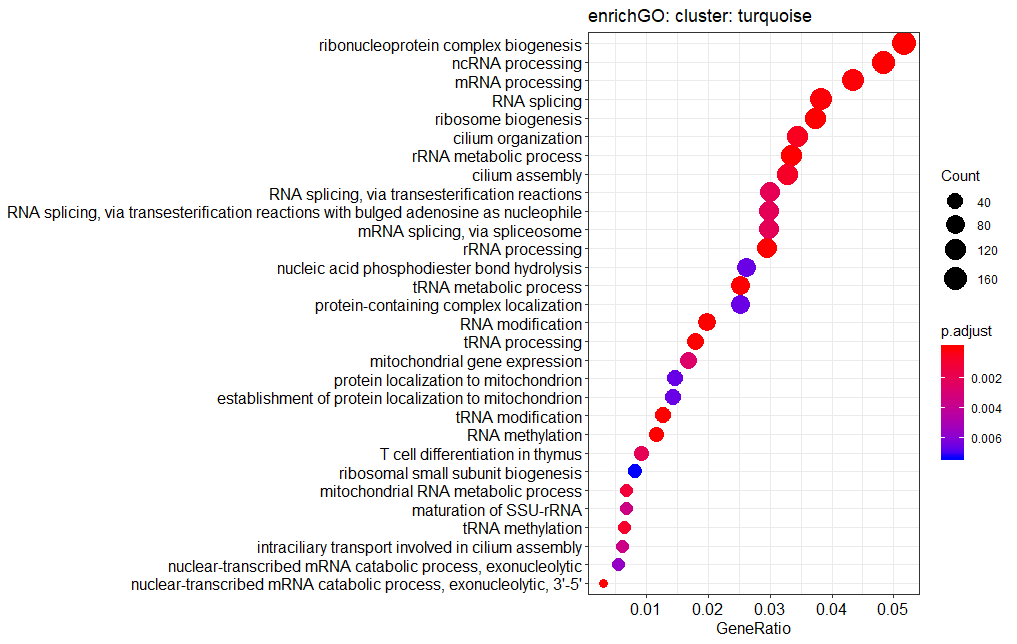


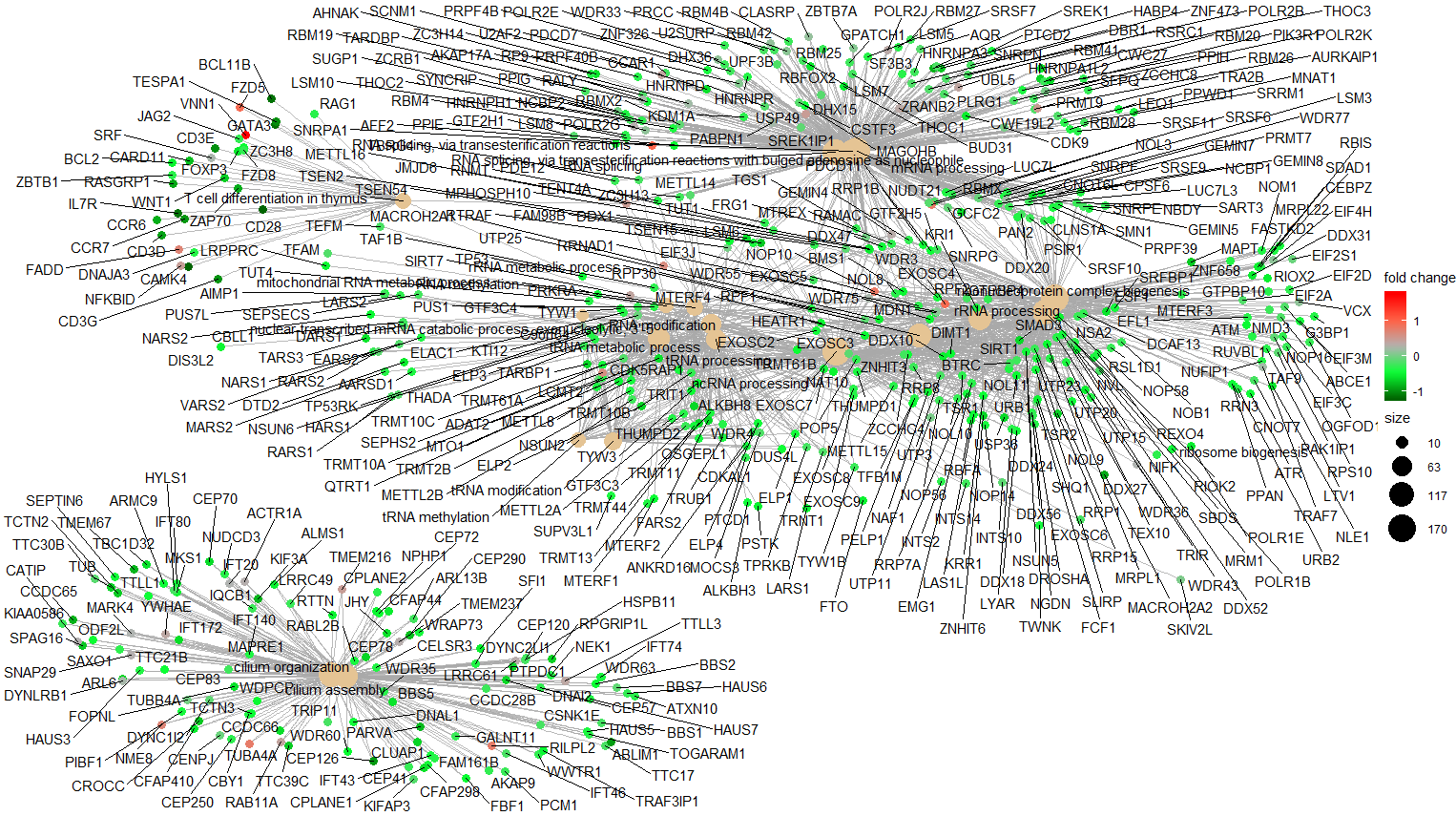


### **B: Cluster blue – neutrophil response**


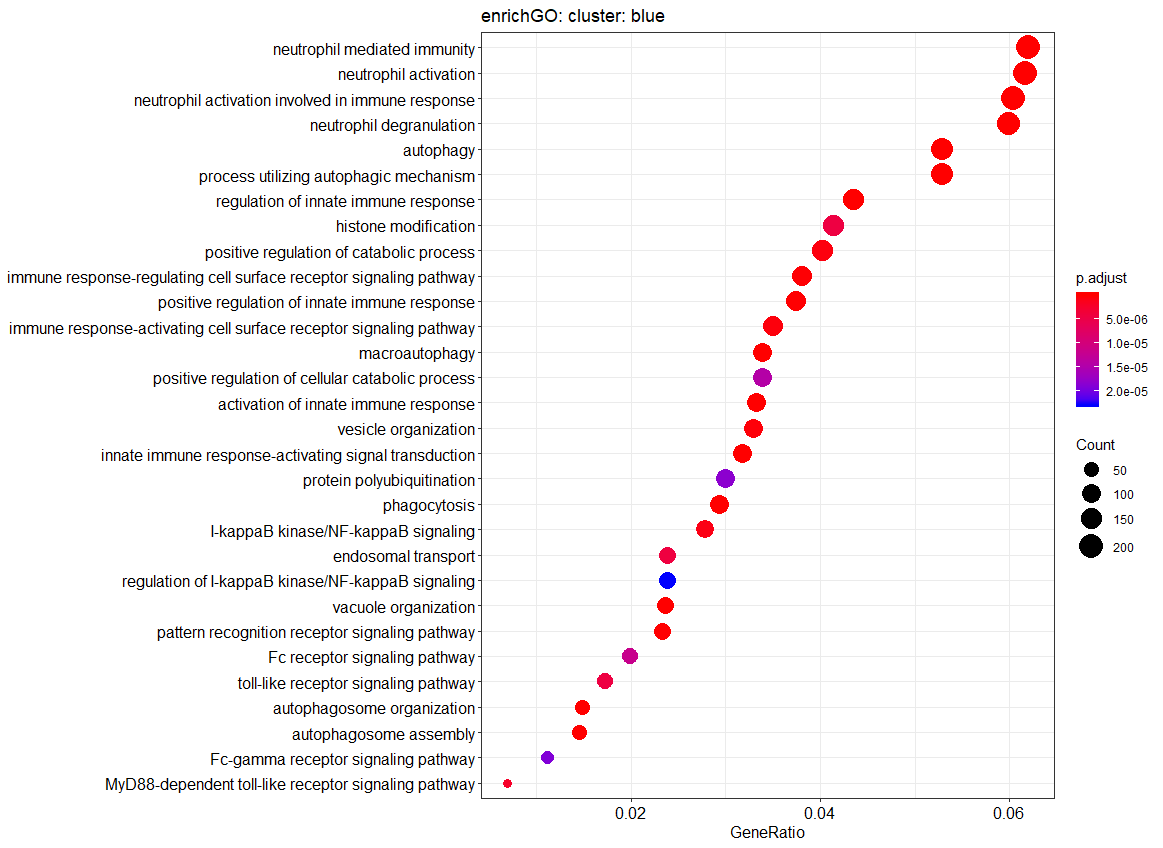


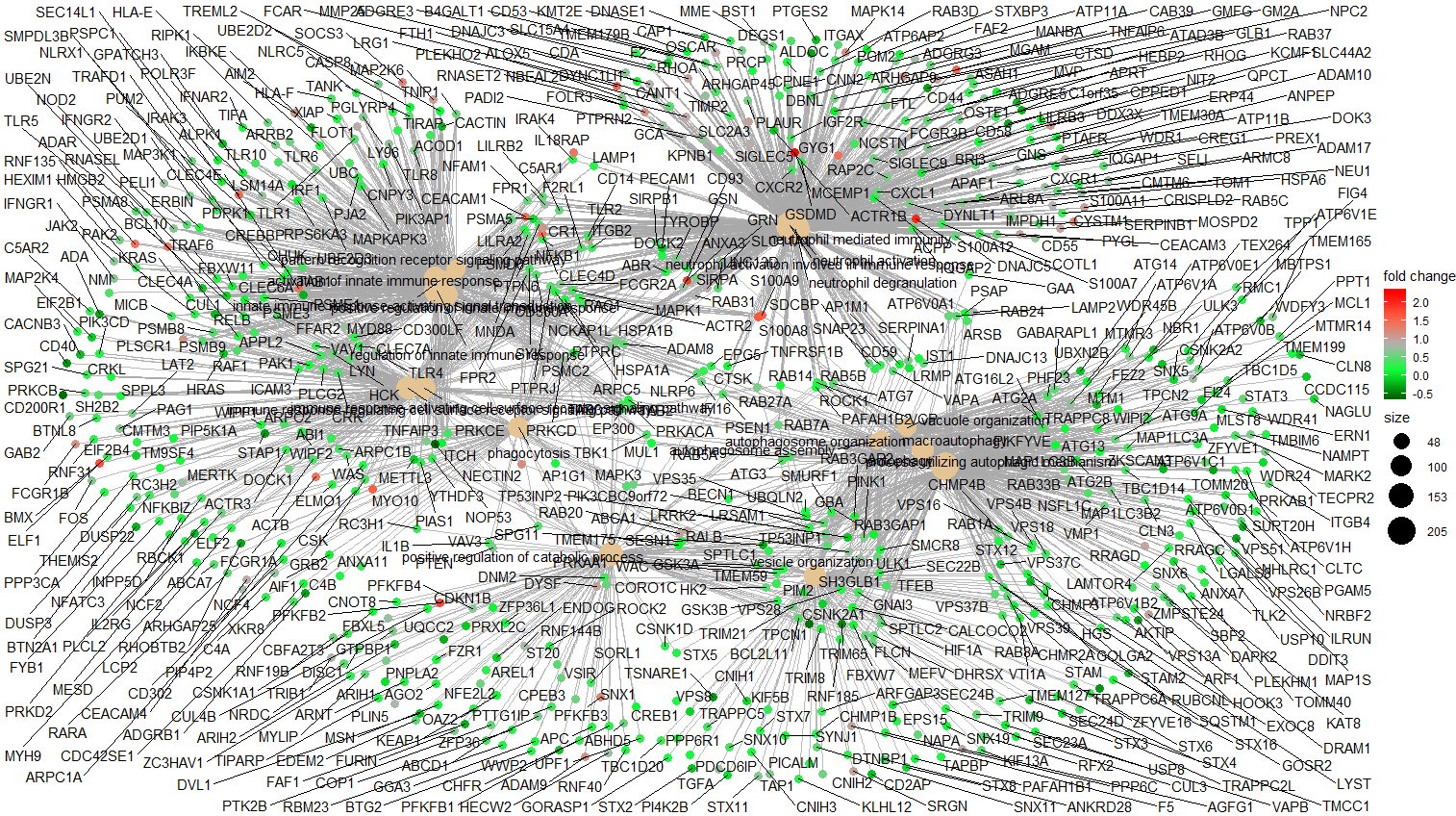


### **C: Cluster brown – sensory perception**


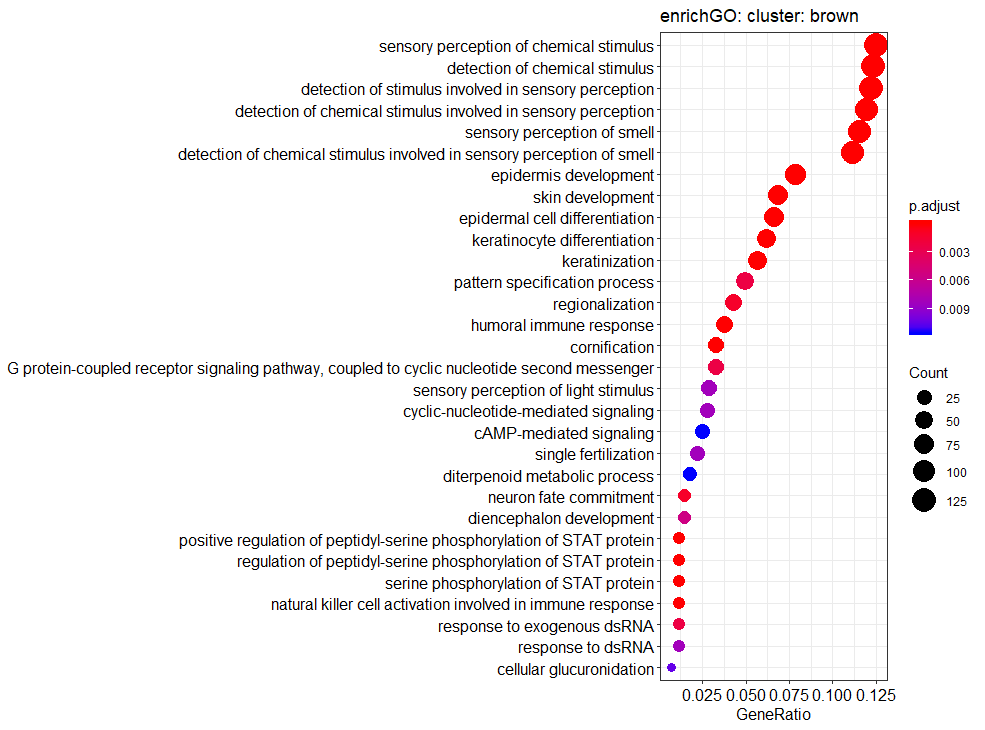


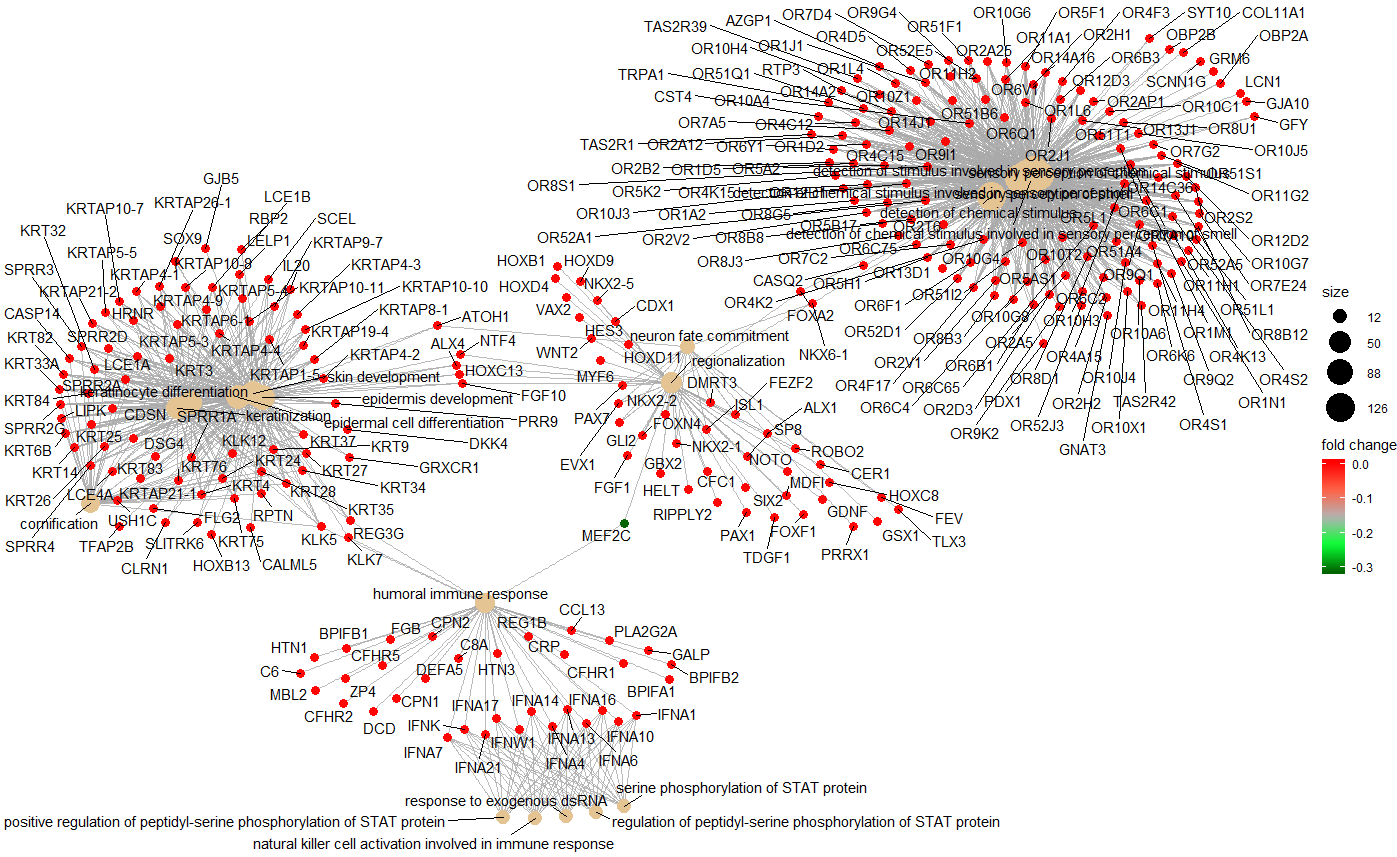


### **D: Cluster yellow – protein DNA complex**


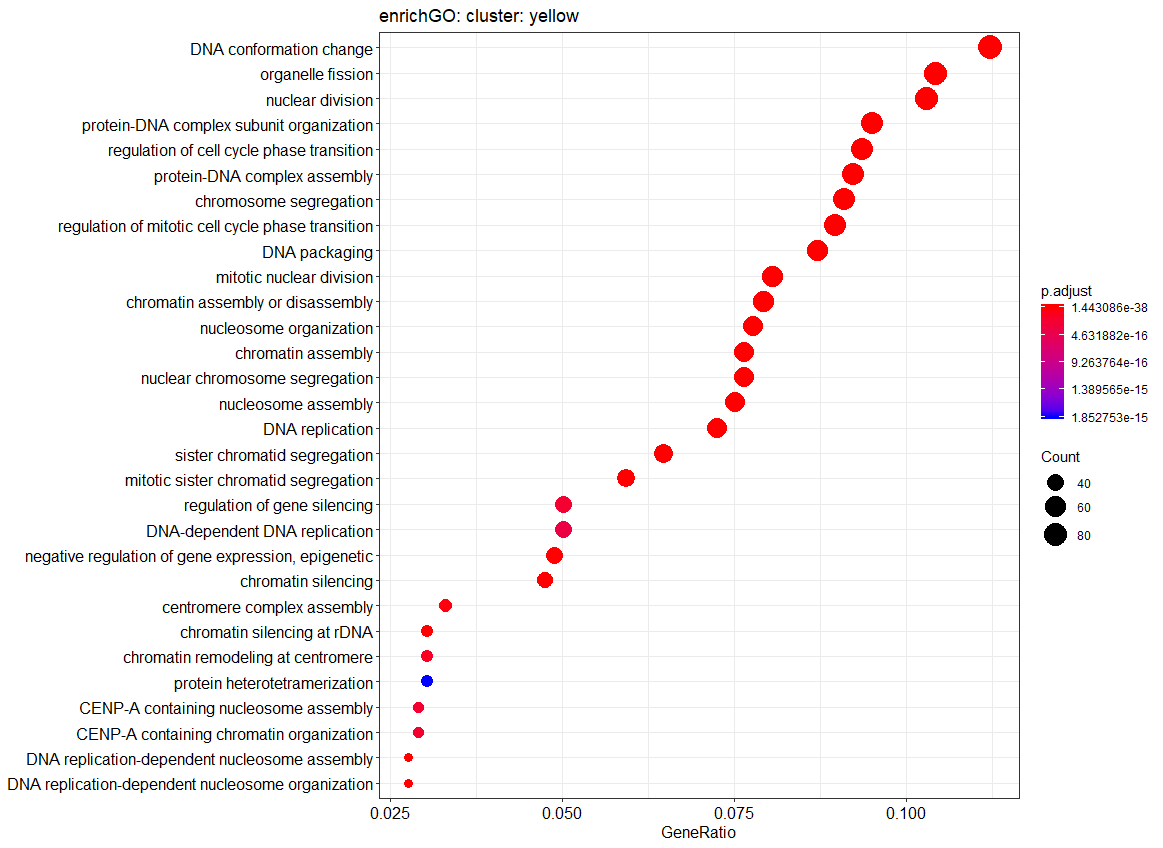


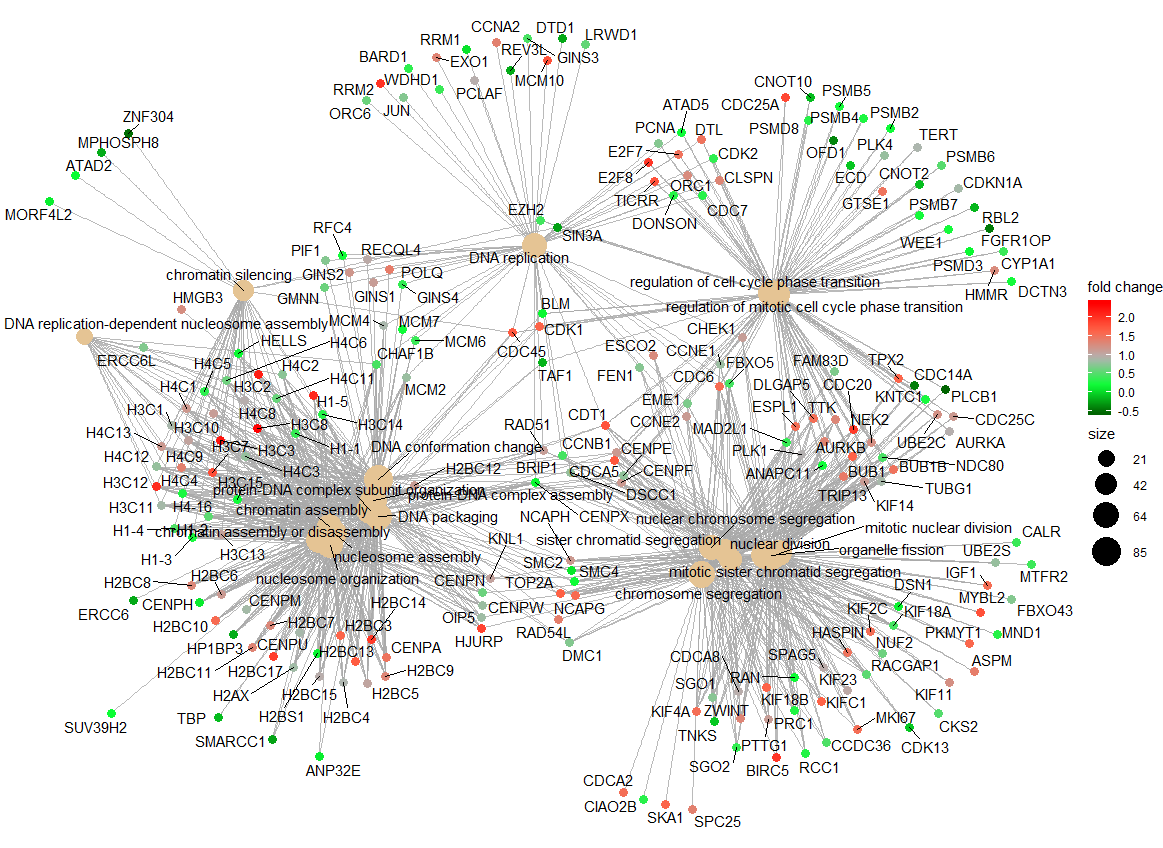


### **E: Cluster green – mRNA processing**


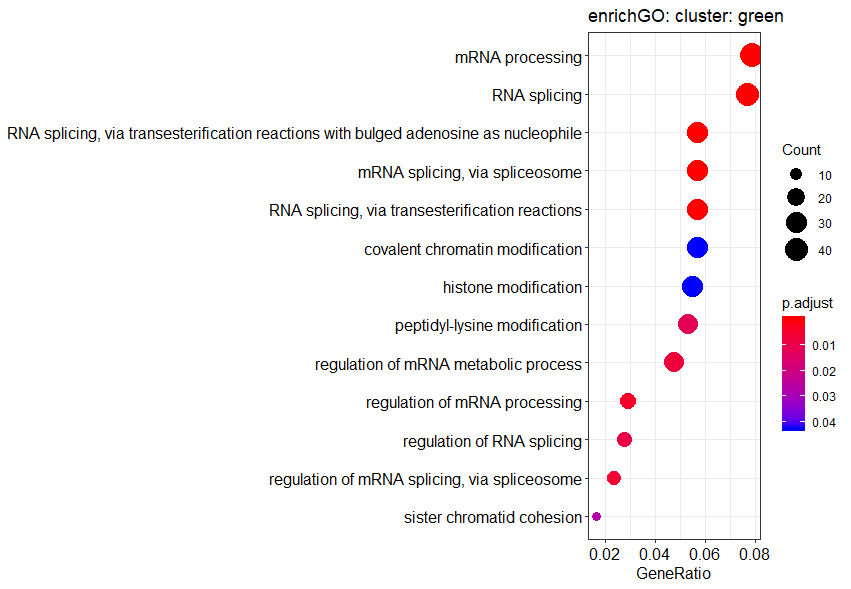


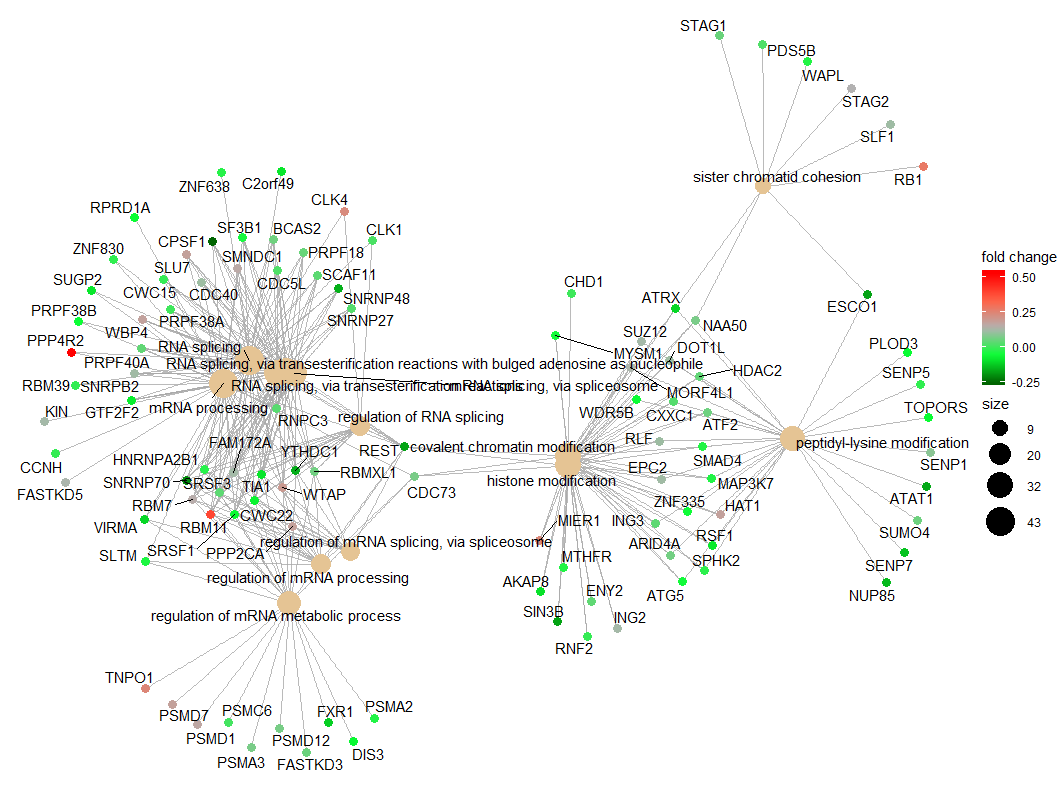


### **F: Cluster red – proteasome catabolic process**


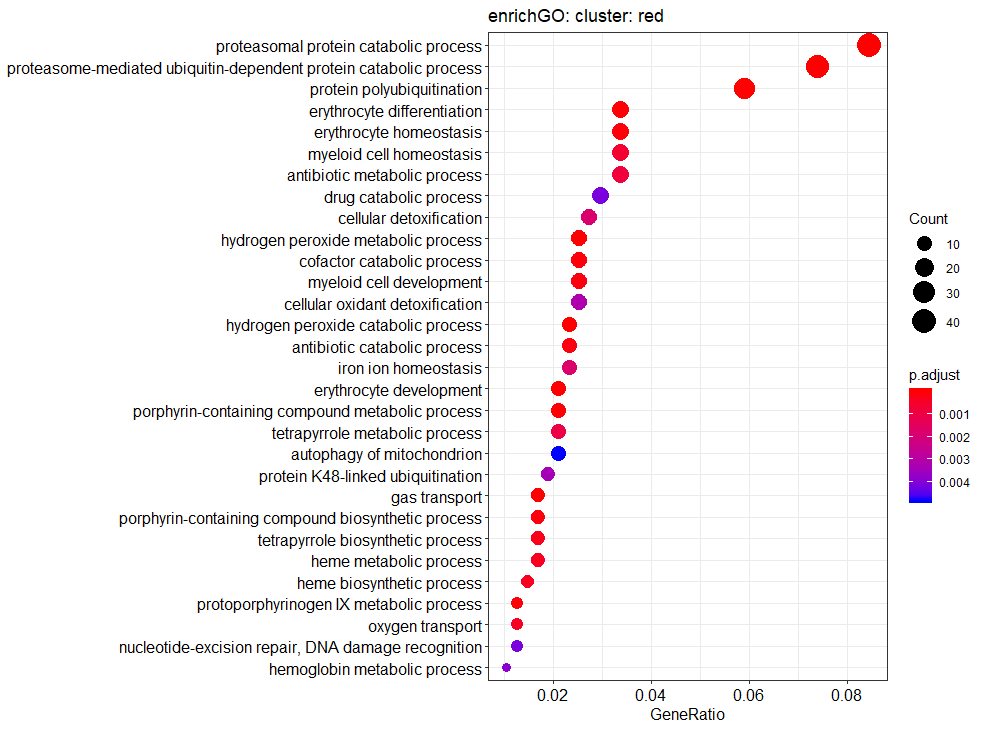


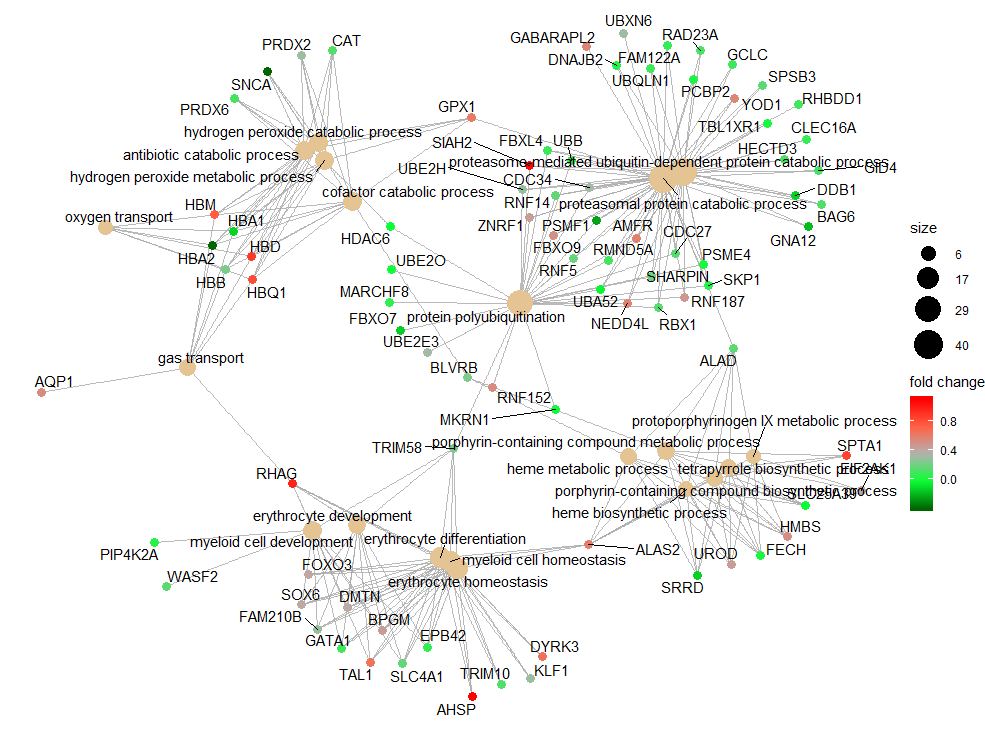


### **G: Cluster black – response to virus**


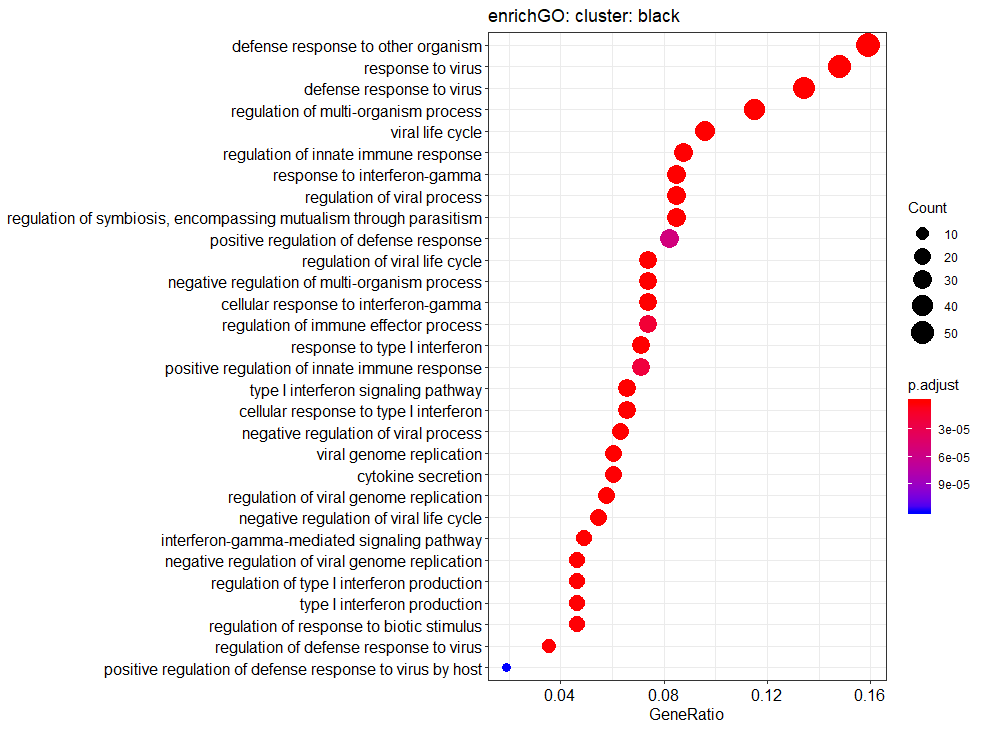


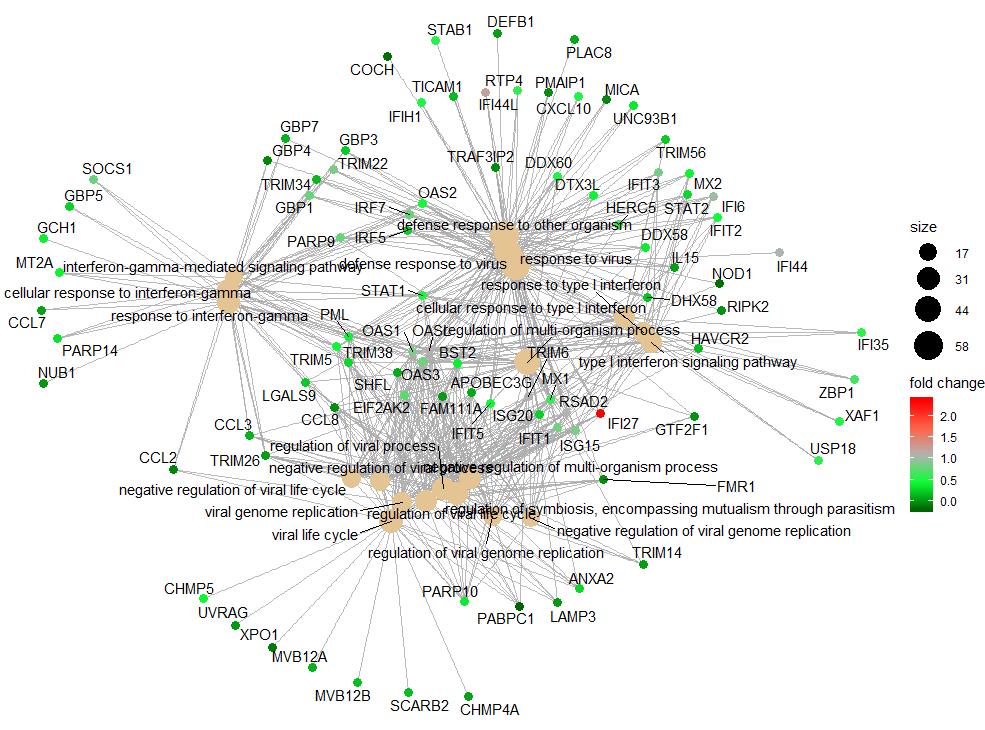


### **H: Cluster pink – blood coagulation**


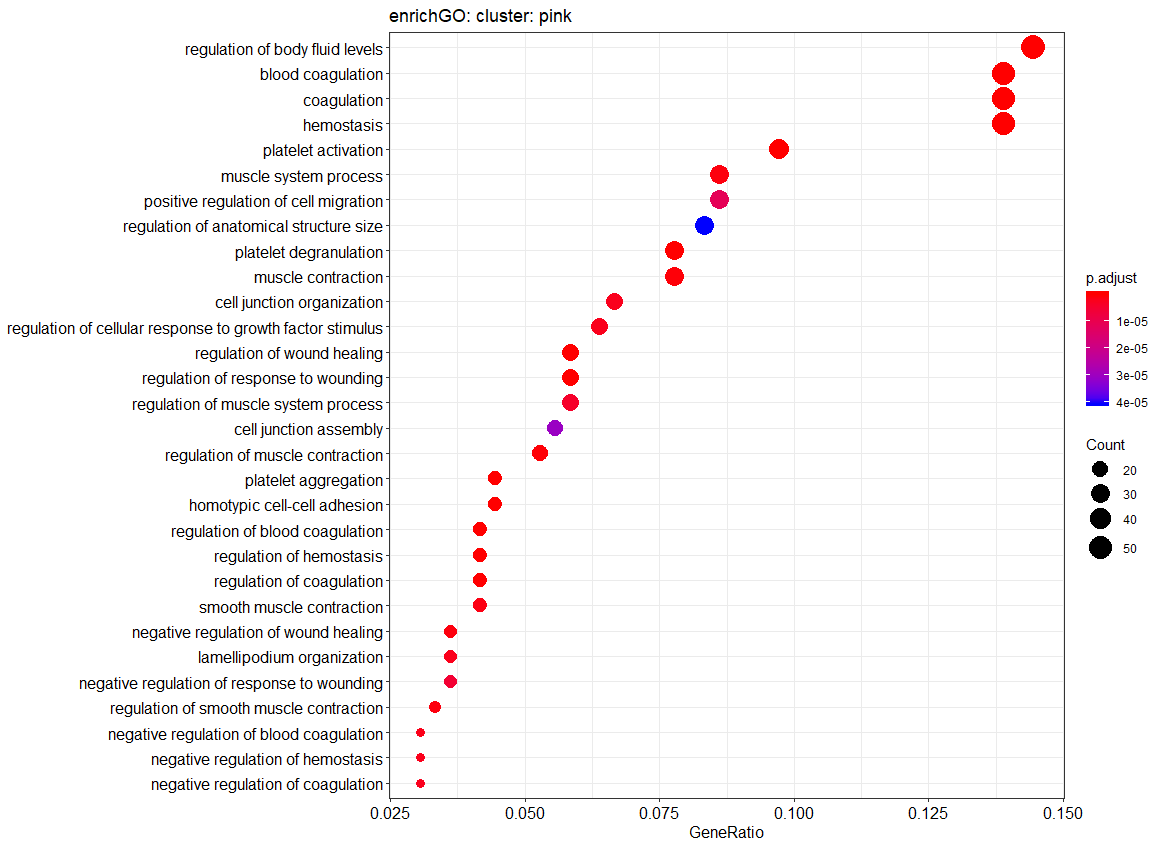


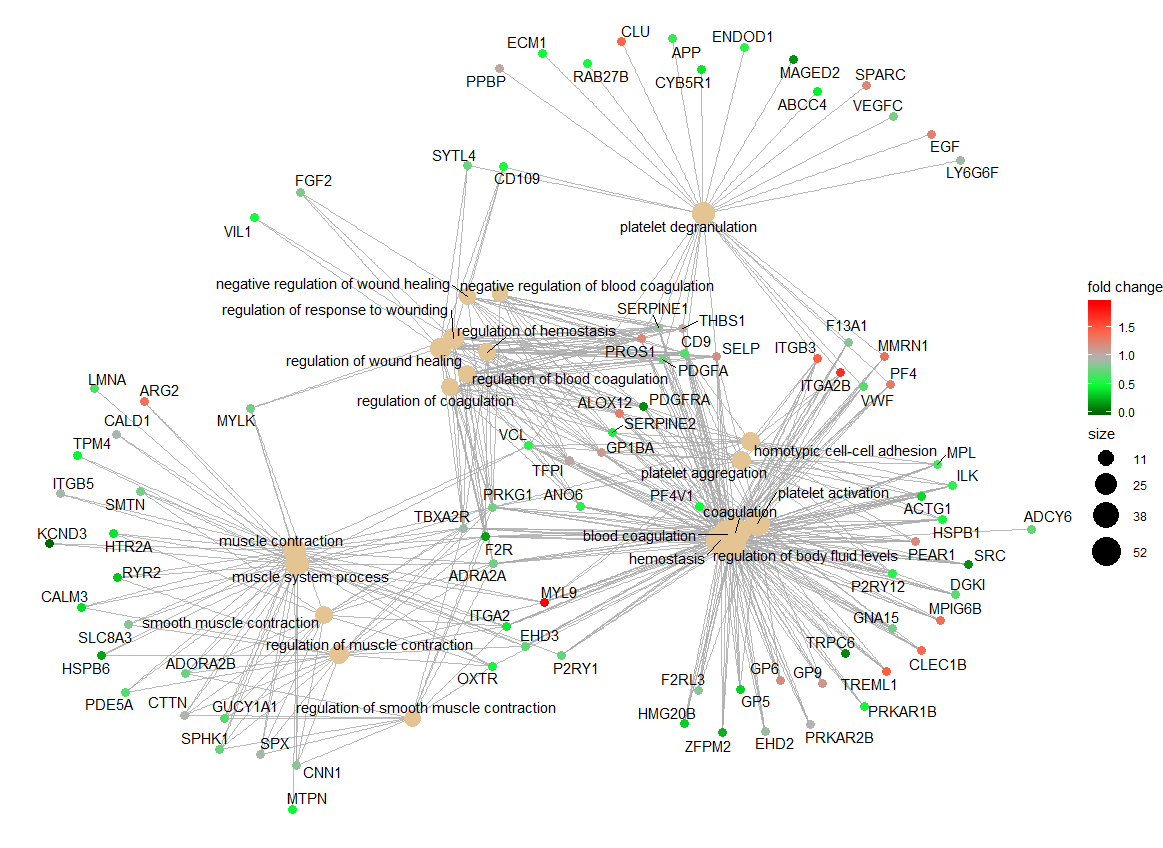


### **J: Cluster magenta – ribonucleoprotein complex**


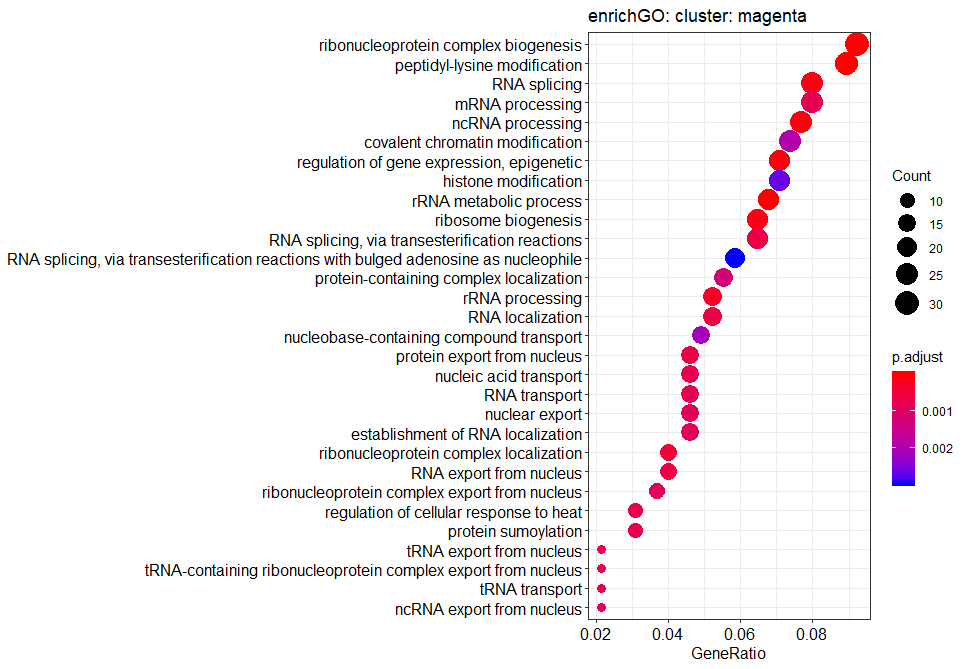


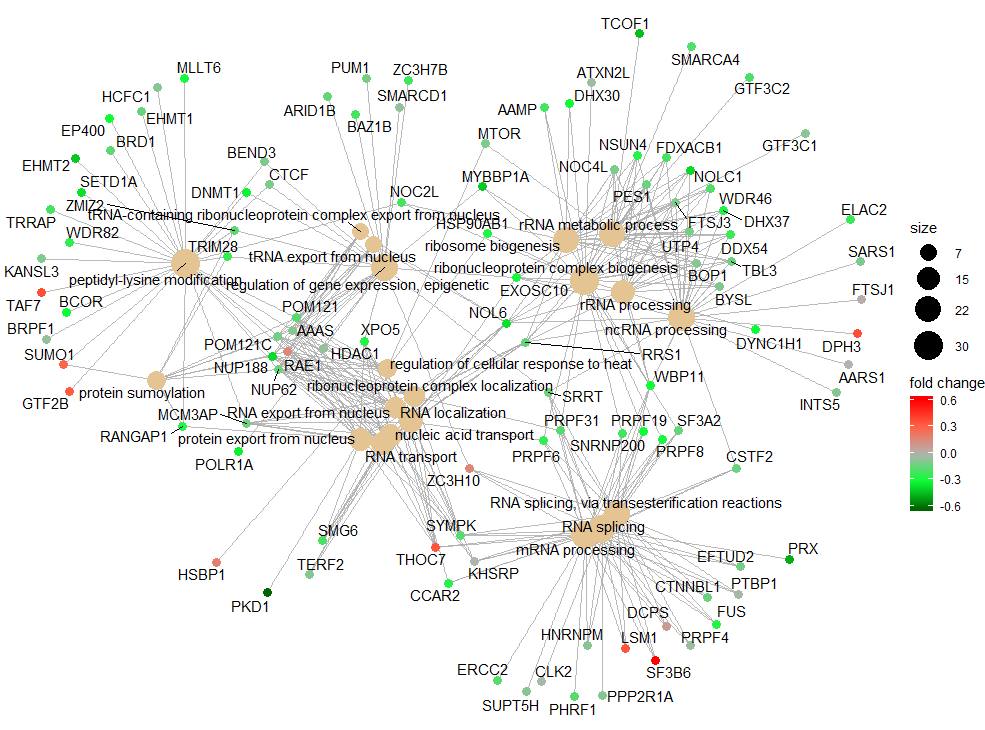


### **K: Cluster purple – lymphocyte differentiation**


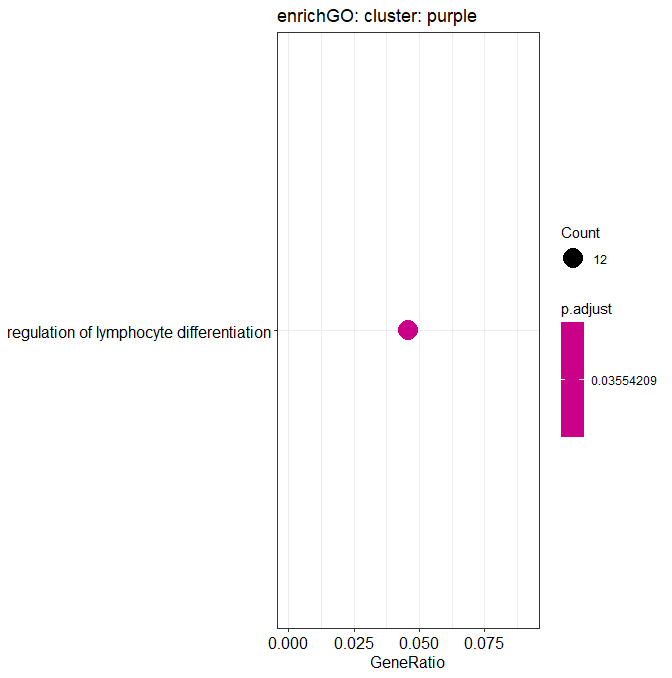


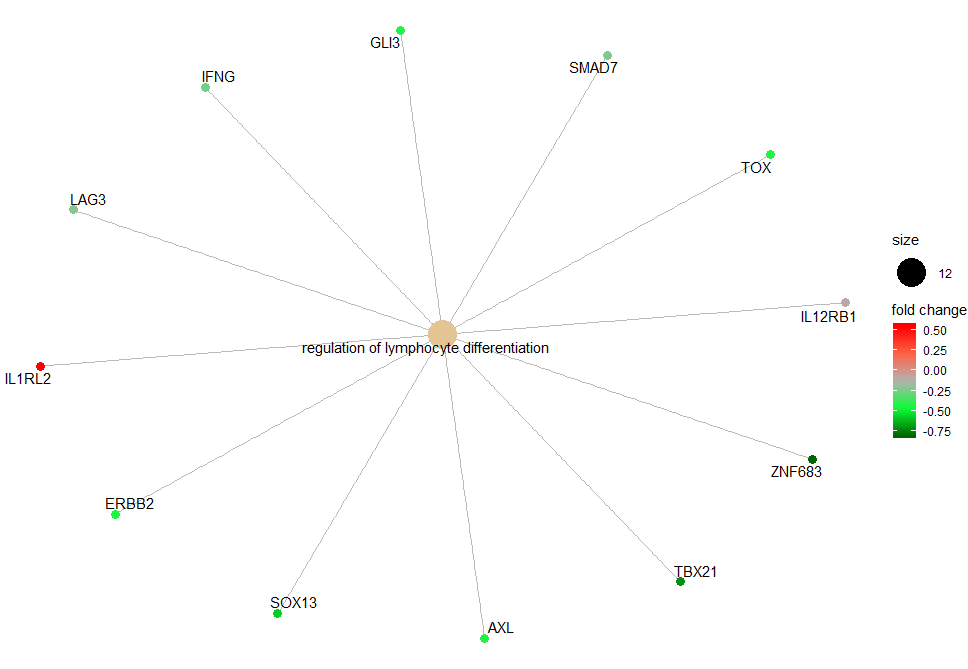


### **L: Cluster greenyellow – neutrophil activation**


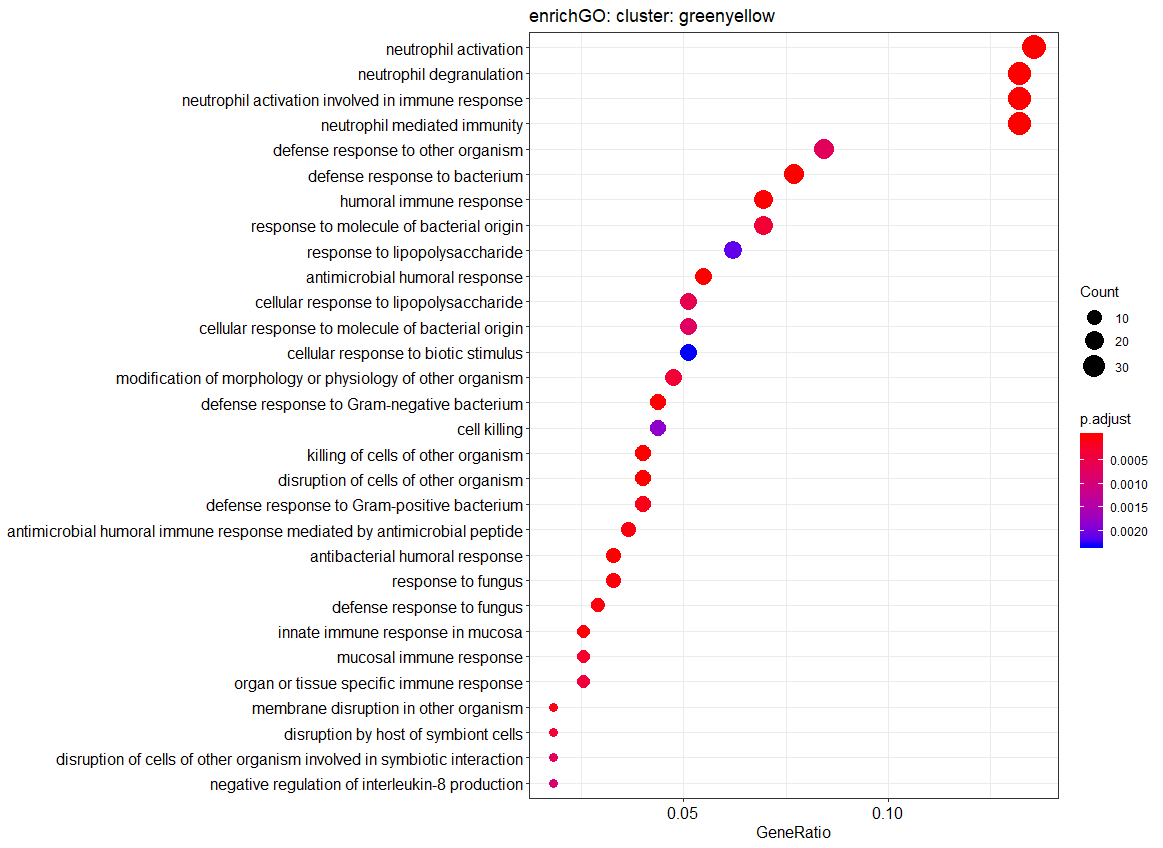


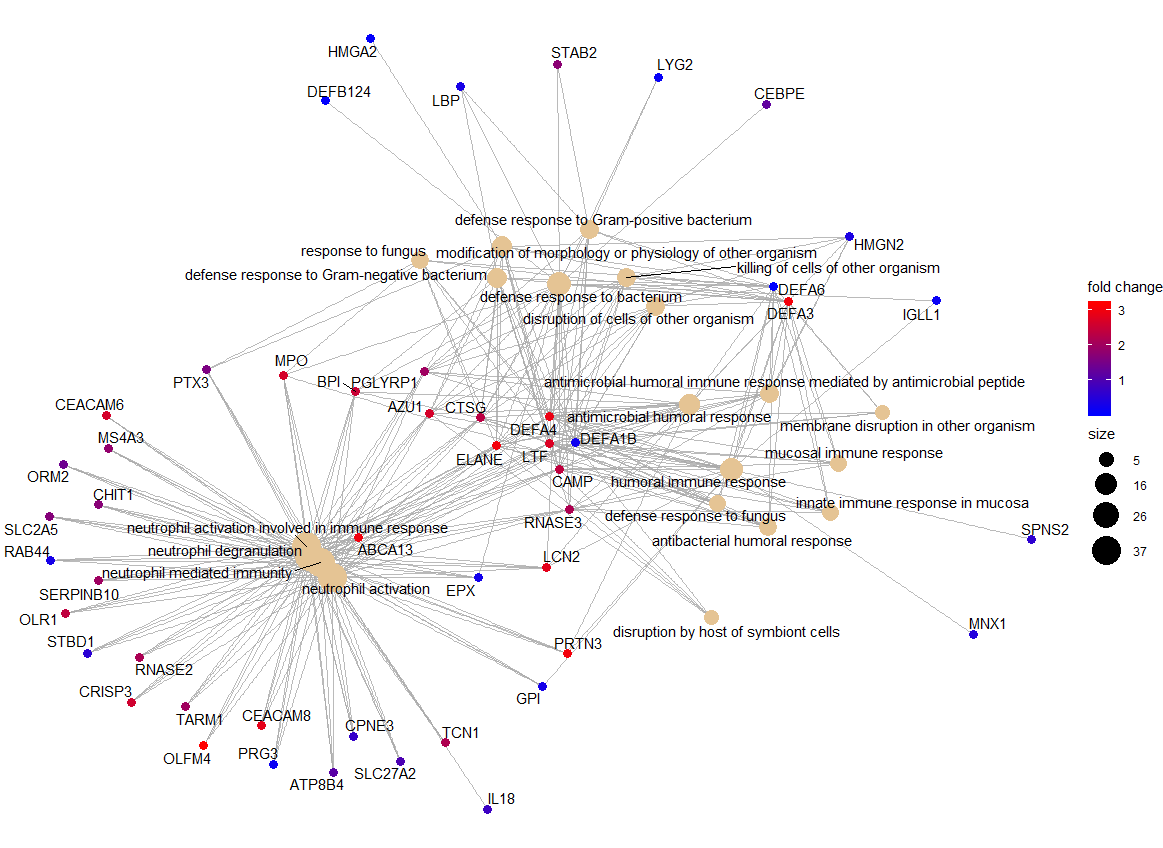


### **M: Cluster tan – B cell activation**


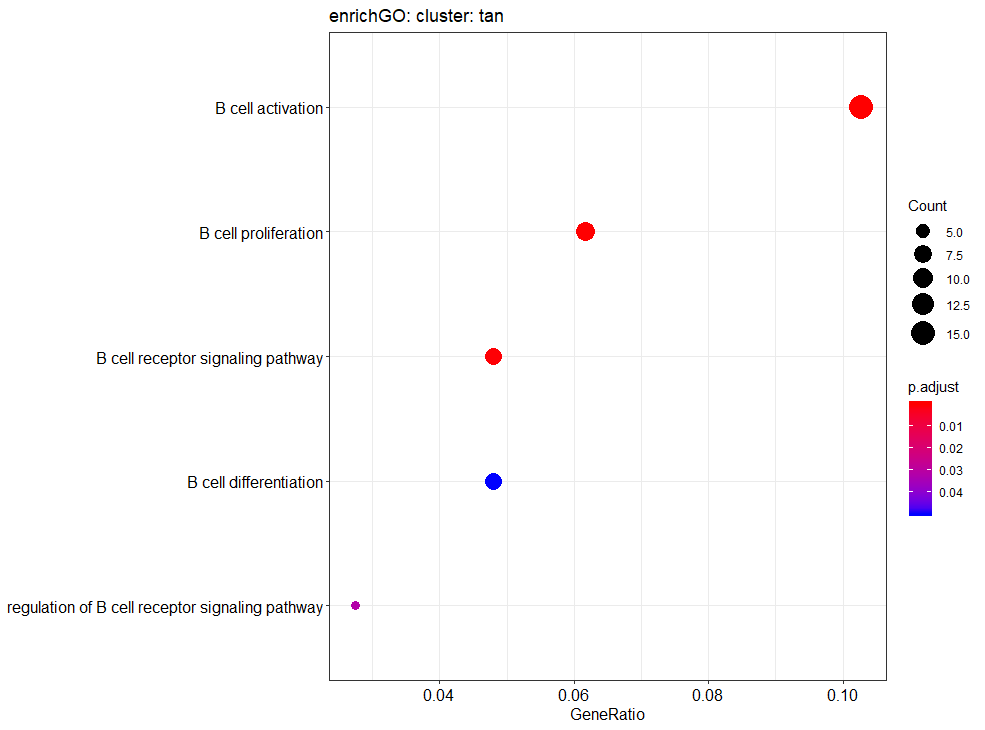


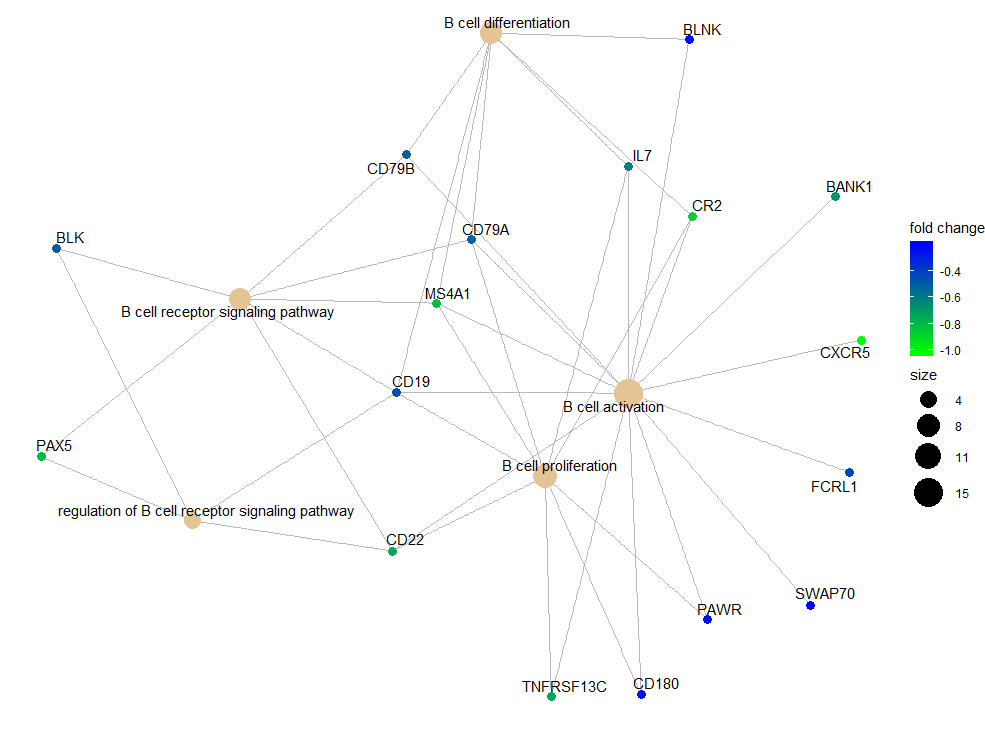


### **N: Cluster salmon – adaptive immune response**


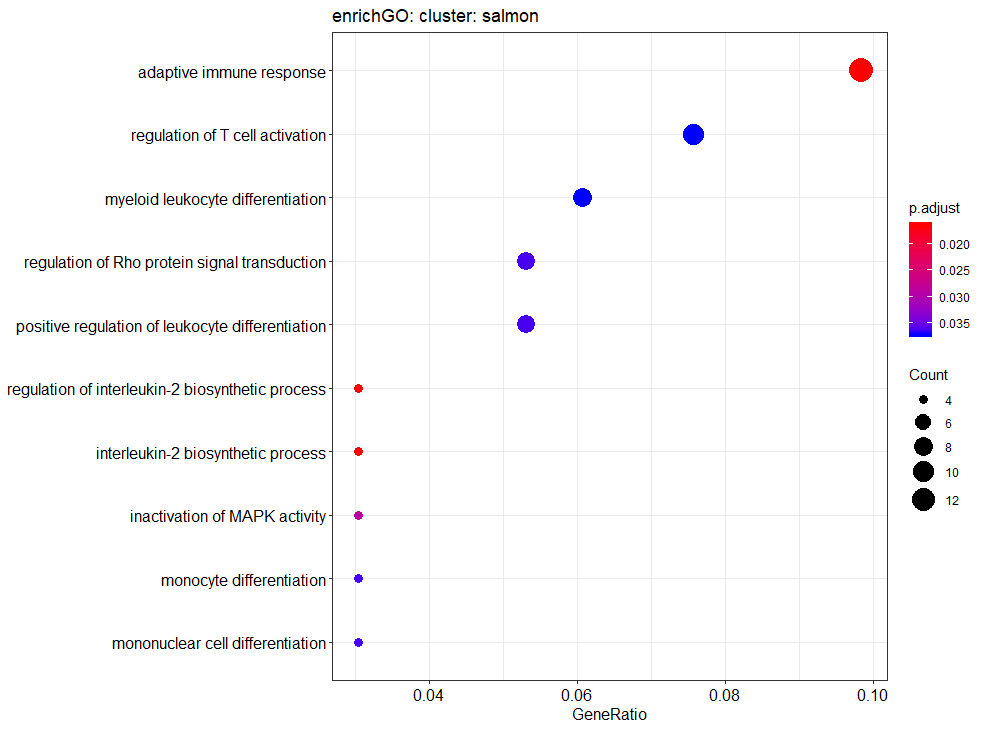


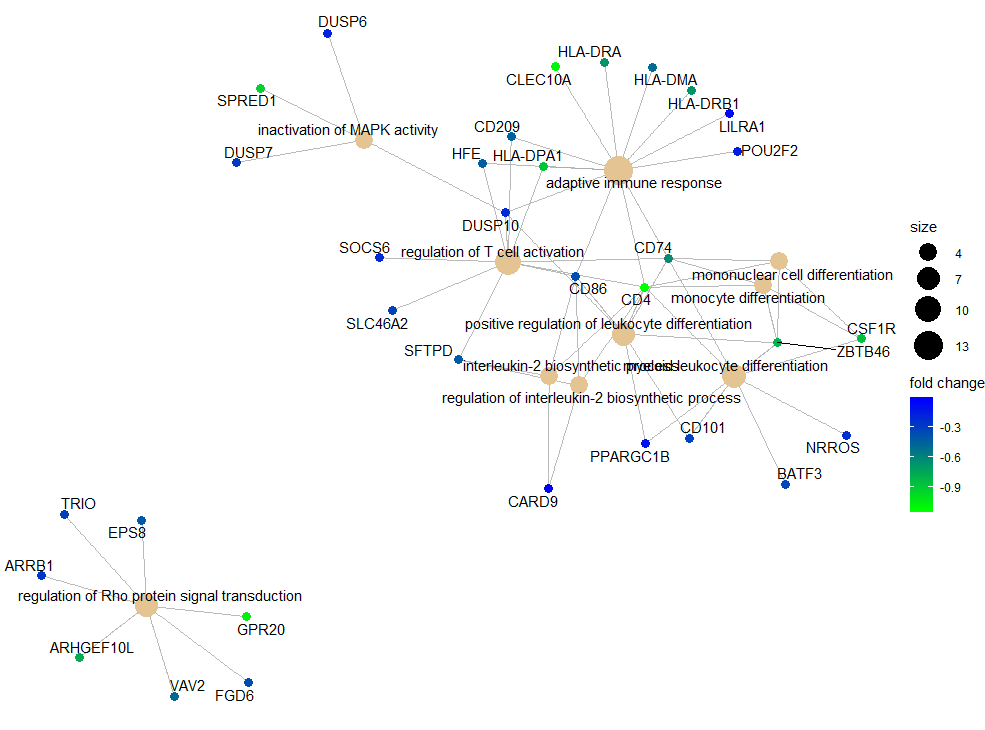


### **O: Cluster cyan – protein targeting to membrane**


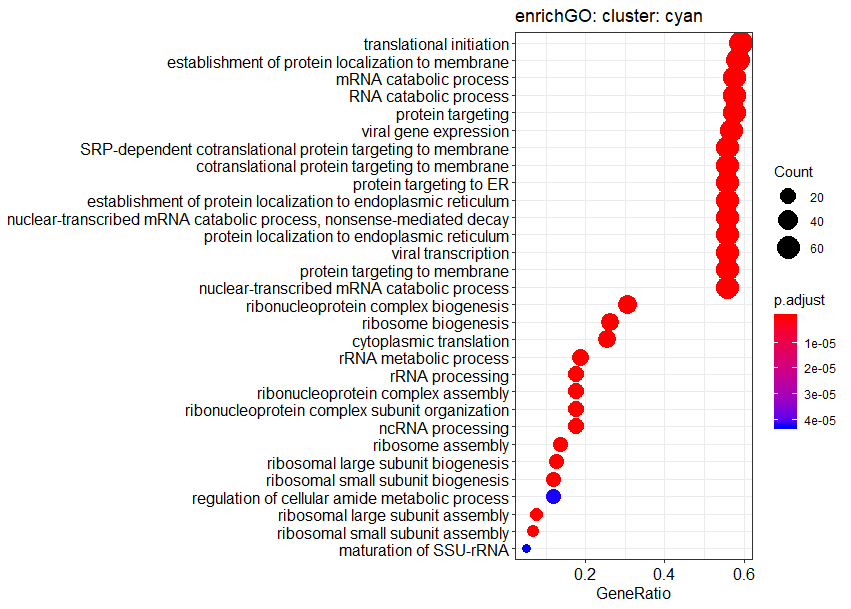


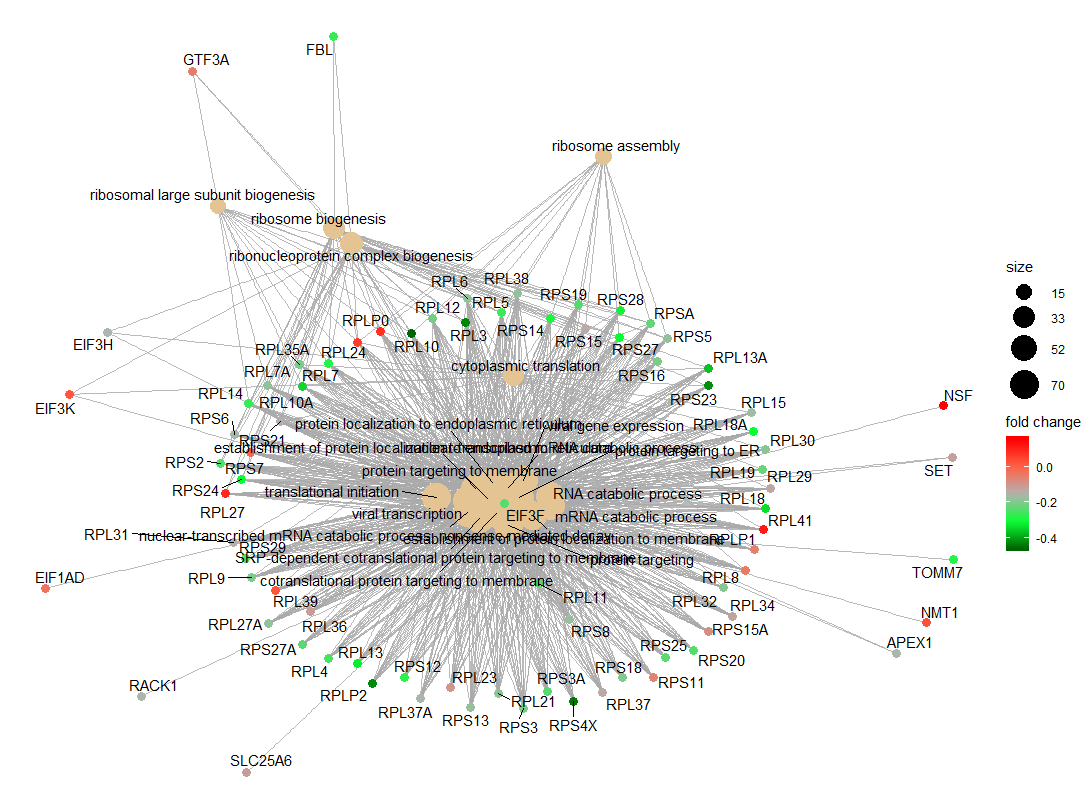


### **P: Cluster midnightblue – no pathway**

No EnrichGO pathway

### **Q: Cluster lightcyan – mRNA processing**


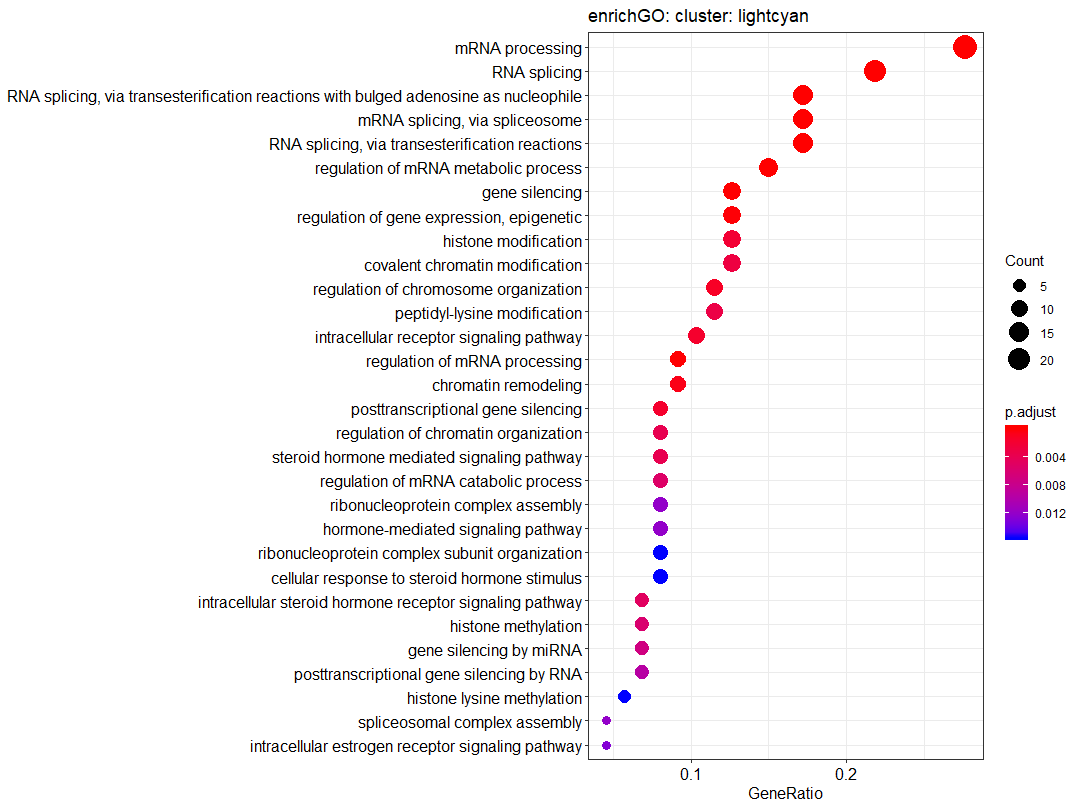


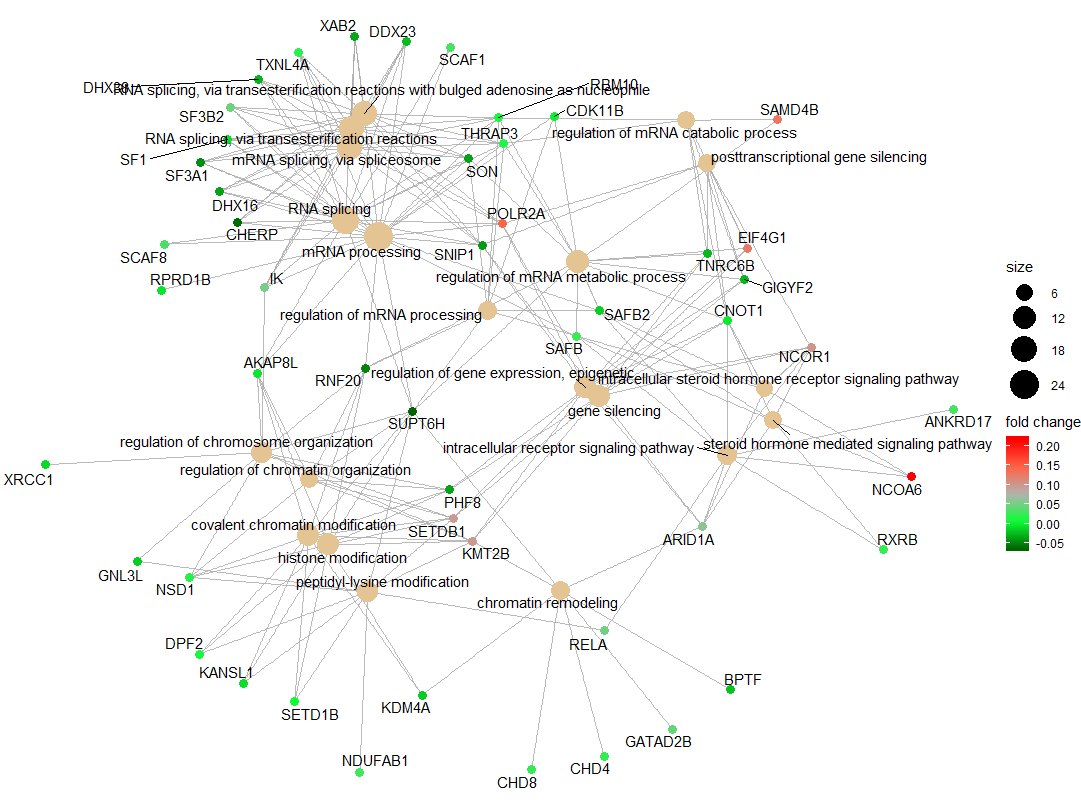


### **R: Cluster grey60 – no pathway**

No EnrichGO pathway

### **S: Cluster lightgreen – epidermis development**


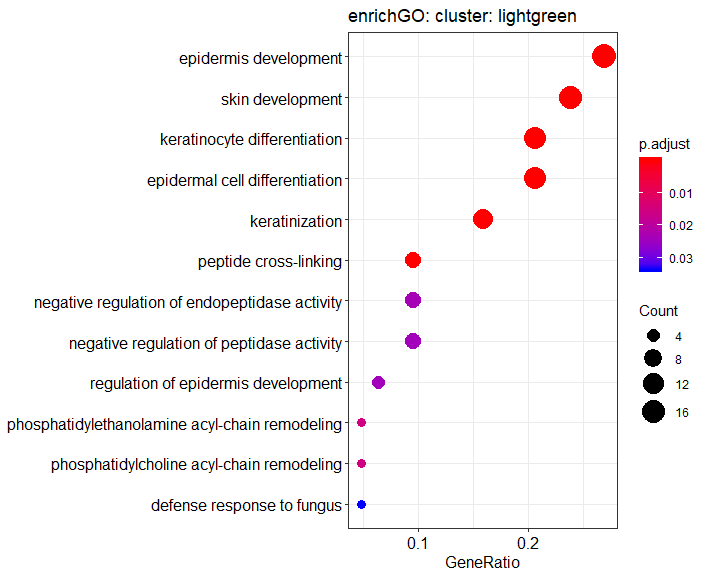


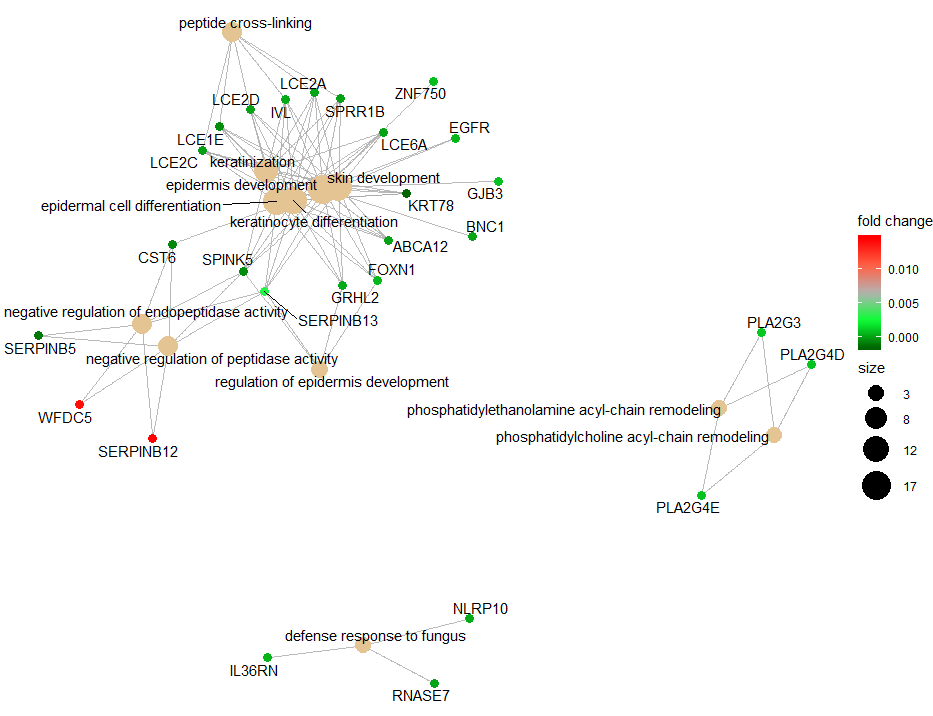


### **T: Cluster lightyellow – no pathway**

No EnrichGO pathway

### **U: Cluster royalblue – no pathway**

No EnrichGO pathway

### **V: Cluster darkred – telomere maintenance**


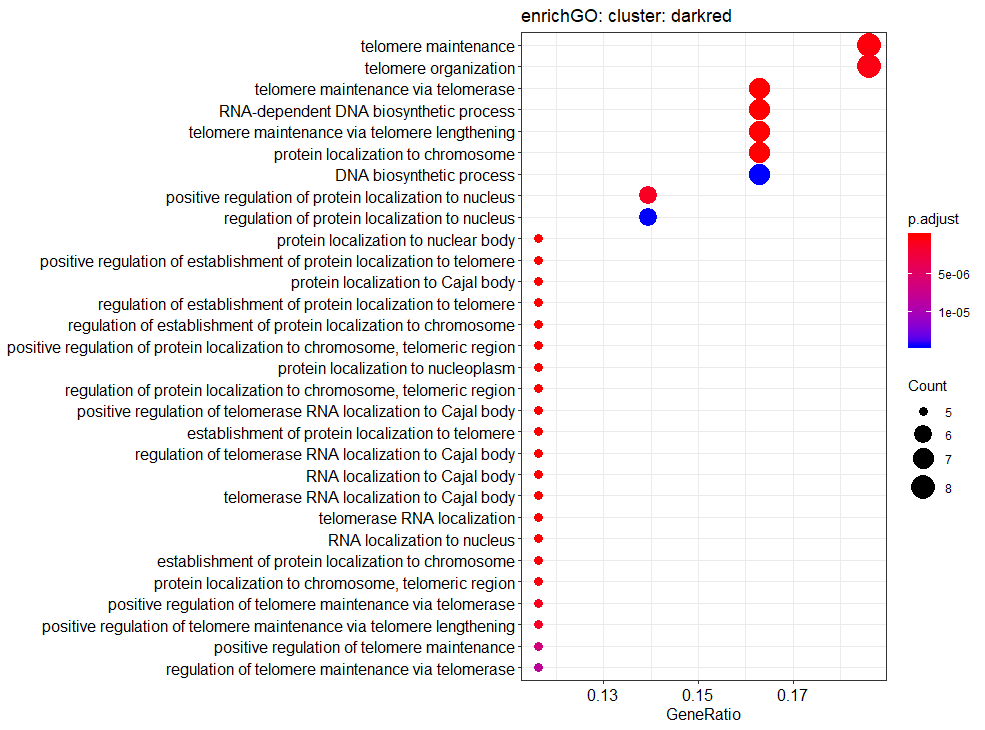


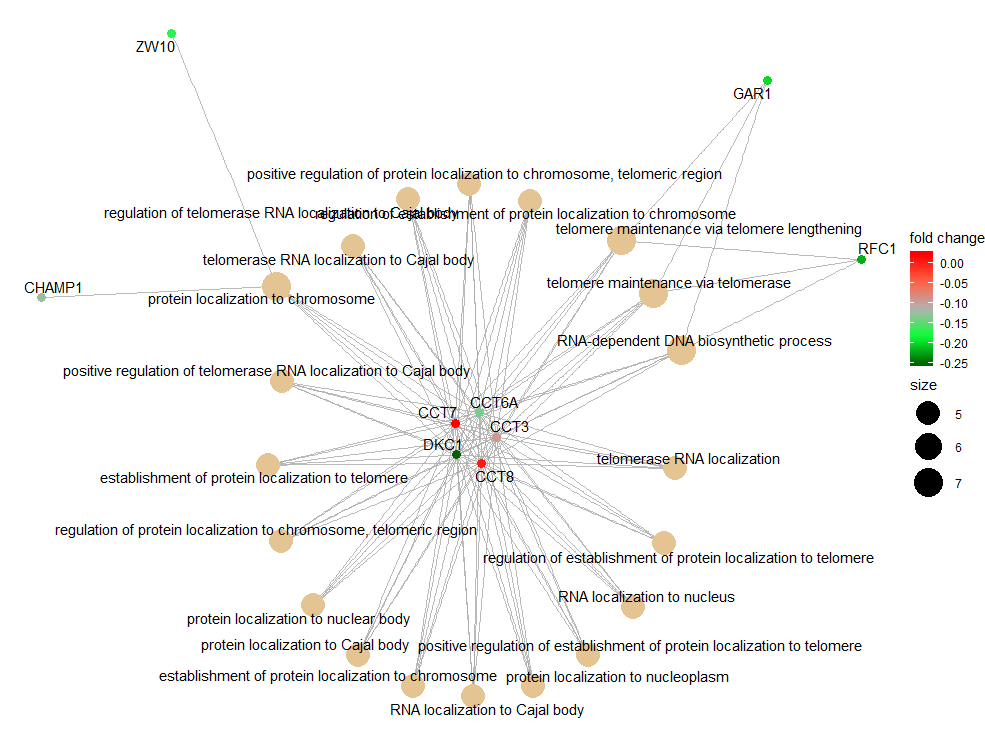

Supplement: Supplementary file 14 [file DataSheet_14.docx]
